# Fibroblast growth factor 10 attenuates chronic obstructive pulmonary disease by protecting against glycocalyx impairment and endothelial apoptosis

**DOI:** 10.1186/s12931-022-02193-5

**Published:** 2022-10-01

**Authors:** Tian Jiang, Weiping Hu, Shaoyuan Zhang, Changhao Ren, Siyun Lin, Zhenyu Zhou, Hao Wu, Jun Yin, Lijie Tan

**Affiliations:** 1 Department of Thoracic Surgery, Zhongshan Hospital, Fudan University, No.180 Fenglin Road, Shanghai, 200032 China; 2 Cancer Center, Zhongshan Hospital, Fudan University, Shanghai, 200032 China; 3 Key Laboratory of Lung Inflammation and Injury, Shanghai, 200032 China; 4 Department of Critical Care and Respiratory Medicine, Zhongshan Hospital, Fudan University, Shanghai, 200032 China; 5 Department of Vascular Surgery, Zhongshan Hospital, Fudan University, Shanghai, 200032 China; 6 Department of Clinical Laboratory Medicine, Zhongshan Hospital, Fudan University, Shanghai, 200032 China

**Keywords:** Chronic obstructive pulmonary diseases (COPD), Fibroblast growth factor 10 (FGF10), Fibroblast growth factor receptor 1 (FGFR1), Glycocalyx, Endothelial apoptosis

## Abstract

**Background:**

The defects and imbalance in lung repair and structural maintenance contribute to the pathogenesis of chronic obstructive pulmonary diseases (COPD), yet the molecular mechanisms that regulate lung repair process are so far incompletely understood. We hypothesized that cigarette smoking causes glycocalyx impairment and endothelial apoptosis in COPD, which could be repaired by the stimulation of fibroblast growth factor 10 (FGF10)/FGF receptor 1 (FGFR1) signaling.

**Methods:**

We used immunostaining (immunohistochemical [IHC] and immunofluorescence [IF]) and enzyme-linked immunosorbent assay (ELISA) to detect the levels of glycocalyx components and endothelial apoptosis in animal models and in patients with COPD. We used the murine emphysema model and in vitro studies to determine the protective and reparative role of FGF10/FGFR1.

**Results:**

Exposure to cigarette smoke caused endothelial glycocalyx impairment and emphysematous changes in murine models and human specimens. Pretreatment of FGF10 attenuated the development of emphysema and the shedding of glycocalyx components induced by CSE in vivo. However, FGF10 did not attenuate the emphysema induced by endothelial-specific killing peptide CGSPGWVRC-GG-_D_(KLAKLAK)_2_. Mechanistically, FGF10 alleviated smoke-induced endothelial apoptosis and glycocalyx repair through FGFR1/ERK/SOX9/HS6ST1 signaling in vitro. FGF10 was shown to repair pulmonary glycocalyx injury and endothelial apoptosis, and attenuate smoke-induced COPD through FGFR1 signaling.

**Conclusions:**

Our results suggest that FGF10 may serve as a potential therapeutic strategy against COPD via endothelial repair and glycocalyx reconstitution.

**Supplementary Information:**

The online version contains supplementary material available at 10.1186/s12931-022-02193-5.

## Highlights

- It is the first time to prove the confirm the endothelial glycocalyx impairment in COPD.
- FGF10 attenuates the development of emphysema and the shedding of glycocalyx induced by CSE in vivo.
- FGF10 alleviates smoke-induced endothelial apoptosis and glycocalyx repair through FGFR1/ERK/SOX9/HS6ST1 signaling.

**Supplementary Information:**

The online version contains supplementary material available at 10.1186/s12931-022-02193-5.

## Introduction

Chronic obstructive pulmonary diseases (COPD), comprised of chronic bronchitis, structural pulmonary alternations, and emphysema, is a progressive life-threatening clinical syndrome with an enormous global burden [1]. The majority of patients with COPD have little therapeutic options at present; therefore, it is imperative to identify and understand the pathophysiology of COPD, which may represent the first step toward the development of novel therapies. The pathological phenotype of COPD and its most important phenotype emphysema, are featured by lung tissue destruction and ensuing alveolar enlargement. On the one hand, pulmonary endothelial apoptosis is critical and maybe prequal to COPD [2], as direct induction of apoptosis in pulmonary endothelial cells causes emphysematous changes [3]. On the other hand, COPD (mostly emphysema) is also considered a disease which is caused by the defects in lung development/repair and structural maintenance [4–6]. Therefore, elucidation of the pathogenetic mechanism underlying impaired lung repair process is key to unravel novel therapeutic target for COPD.

The endothelial glycocalyx is an extracellular layer lining the luminal surface of endothelial cells of the vessels, which maintains vascular homeostasis, regulates vascular barrier permeability, and inhibits intravascular thrombosis [7, 8]. The endothelial glycocalyx is mainly composed of glycoproteins, glycosaminoglycans (heparan sulphate [HS] and chondroitin sulphate [CS]), and syndecan family (syndecan-1 and syndecan-4) [9]. Recent works have highlighted a critical role of endothelial glycocalyx in lung repair, as (1) the endothelial glycocalyx was degraded, releasing highly-sulfate heparin sulfate octa-saccharides into the circulation during indirect lung injury [10]; (2) the cremasteric endothelial glycocalyx thickness recovered after 72 h of acute degradation of the glycocalyx with TNF-α injection [11]; (3) enhancing the sepsis-inhibitory glycocalyx-repairing signal was a potential approach to reconstitute the glycocalyx layer and recover its function [12].

The reparative fibroblast growth factor receptor (FGFR) signaling is an evolutionary conserved signaling cascade that regulates several basic biologic processes in lungs, including embryonic development and tissue regeneration [13, 14]. Yang and colleagues investigated the endogenous mechanisms underlying homeostatic pulmonary glycocalyx reconstitution, and identifies FGFR1 as a critical mediator of glycocalyx repair and is suppressed during sepsis [10]. In addition, FGF-FGFR1 axis was reported to play an autocrine role during vascular remodeling in COPD [15]. Fibroblast growth factor 10 (FGF10) is critical for lung development and renewal [16, 17], with its essential role illustrated by the complete failure of lung formation and perinatal lethality in *FGF10* deficient mice [17–19], and its reparative capability in different types of lung injury and disease of animal models [20]. Importantly, FGF10 signaling is dysregulated in various human lung diseases including COPD [21], as heterozygous loss-of-function mutations in FGF10 causes COPD in human [22]. FGF10 acts as a ligand for both FGFR1b and FGFR2b [19]. Given that FGFR1 is the predominant receptor of FGF family expressed in pulmonary endothelial cells [23] and the important role of FGF10 in lung tissue repair, we hypothesize that stimulation of FGF10/FGFR1 signaling could repair smoke-induced pulmonary glycocalyx injury and endothelial apoptosis, and attenuate COPD and emphysema.

## Methods and materials

### Patient samples

Human lung sections were obtained from 10 COPD patients and compared to 7 non-COPD donors who underwent lung cancer resection at our institute. The lung tissue 5 cm away from tumor margin was used in our studies. Blood samples from 14 COPD and 25 non-COPD patients were collected using a serum separator tube (SST, Becton Dickinson Labware, Franklin Lakes, NJ) and immediately centrifuged at 3000*g* 4 °C for 10 min. The serums were subsequently removed and stored at − 80 °C for further analysis. All patients did not receive radiotherapy, chemotherapy or lung transplant, and did not have any other confoundable medical conditions. Lung function tests were conducted in all subjects by standardized methods according to the American Thoracic Society guidelines [24]. COPD was defined based on the revised GOLD 2017 COPD categorization [25]. Spirometry parameters (FEV1/FVC and FEV1% predicted) and other patient characteristics were collected and summarized in Additional file 1: Table S1. The study protocol conforms to the principles of the Declaration of Helsinki and was conducted with approval from the Ethics Committee of Zhongshan Hospital at Fudan University in Shanghai, China. All patients signed an informed consent form.

### Animal models

All animals were purchased from the Shanghai SLAC laboratory animal Co. Ltd. (Shanghai, China), and animal experimental protocols were approved by the Ethics Committee of Fudan University, and all experiments were performed in accordance with the “Guide for the Care and Use of Laboratory Animals” (Institute of Laboratory Animal Resources, 7th edition 1996).

We set several animal experiments as follows: *(1)* Cigarette smoke-induced mouse COPD model: To establish the cigarette smoke-induced COPD model, the male C57BL/6J mice (6–8 weeks; ~ 20 g) were exposed to the cigarette smoke or air using a whole-body smoking exposure apparatus (Data Sciences International Co., USA). For the cigarette smoke group, mice were placed in the smoking exposure apparatus and exposed to 10 filter-tipped cigarettes twice a day, 5 days per week, for 1 month or 3 months. Each cigarette smoke exposure duration lasted 1 h with an interval time between two exposures more than 4 h. *(2)* Rat emphysema model: To induce the emphysema formation in animals, the pan-vascular endothelial growth factor-receptor (VEGF-R) inhibitor SU5416 (20 mg/kg; MedChemExpress) suspended in solution (10% DMF mixed with 90% coin-oil; both obtained from MedChemExpress) was injected subcutaneously 3 times per week for 3 weeks. *(3)* Cigarette Smoke Extract (CSE)-induced mouse emphysema model: The emphysema mouse model was built according to the protocol of the previous study [26]. Male C57BL/6 mice (6–8 weeks; ~ 20 g) received 400 μl CSE intraperitoneally at Days 1, 8, 15 and 22. FGF10 was administered intravenously 24 h before CSE injection (Day 0, 7, 14 and 21). All mice were sacrificed at Day 28. The doses of FGF10 for the emphysema model were selected based on previous experiments [27–29]. The treatments were as follows: (1) Control group; (2) CSE group; (3) CSE + FGF10 10 μg/kg group; (4) CSE + FGF10 100 μg/kg group; (5) CSE + FGF10 1 mg/kg; (6) CSE + FGF10 2.5 mg/kg. *(4)* Killing peptide-induced mouse emphysema model: The endothelial cell (EC)-specific killing peptide CGSPGWVRC-GG-_D_(KLAKLAK)_2_ (hereinafter called the ‘KLAKLAK_2_’) was synthesized commercially (Sangon Biotech, Shanghai) and served as positive controls [30]. Male C57BL/6 mice (6–8 weeks; ~ 20 g) were administered standard saline solution, or 240 μg of KLAKLAK_2_ intraperitoneally at Days 1, 8, 15 and 22, and FGF10 intravenously 24 h prior to each injection of CSE at Day 0, 7, 14 and 21. According to the results of emphysema mouse model, mice were randomly divided into four groups (n = 8–10 each), and the treatments were as follows: (1) Control group; (2) KLAKLAK_2_ group; (3) KLAKLAK_2_ + FGF10 1 mg/kg; (4) KLAKLAK_2_ + FGF10 2.5 mg/kg.

### Cell culture

Human pulmonary microvascular endothelial cells (hPMVECs, passages 4–6, Cat #C-12281, PromoCell, Heidelberg, Germany) were used in this study. Cells were treated with different concentrations of cigarette smoke extract (CSE) (Additional file 1: Figure S1), FGF10 (R&D Systems, Minneapolis, MN), and FGFR1 inhibitor AZD4547 (MedChemExpress).

### Statistical analysis

Data are expressed as the mean ± standard deviation (SD) and were compared by Mann–Whitney U-test and Kruskal–Wallis test. Correlation between two groups was analyzed using Pearson correlation test. Statistical significance was assumed at p < 0.05. SPSS (Version 24.0; IBM, Armonk, NY, USA) was used to analyze the data. The number of experiments or animals of each group is reported in the figure legend.

Methods are described in detail in Additional file 1: Methods.

## Results

### Exposure to smoking cigarette causes endothelial apoptosis, glycocalyx impairment and emphysematous changes in animal models

**Fig. 1:**
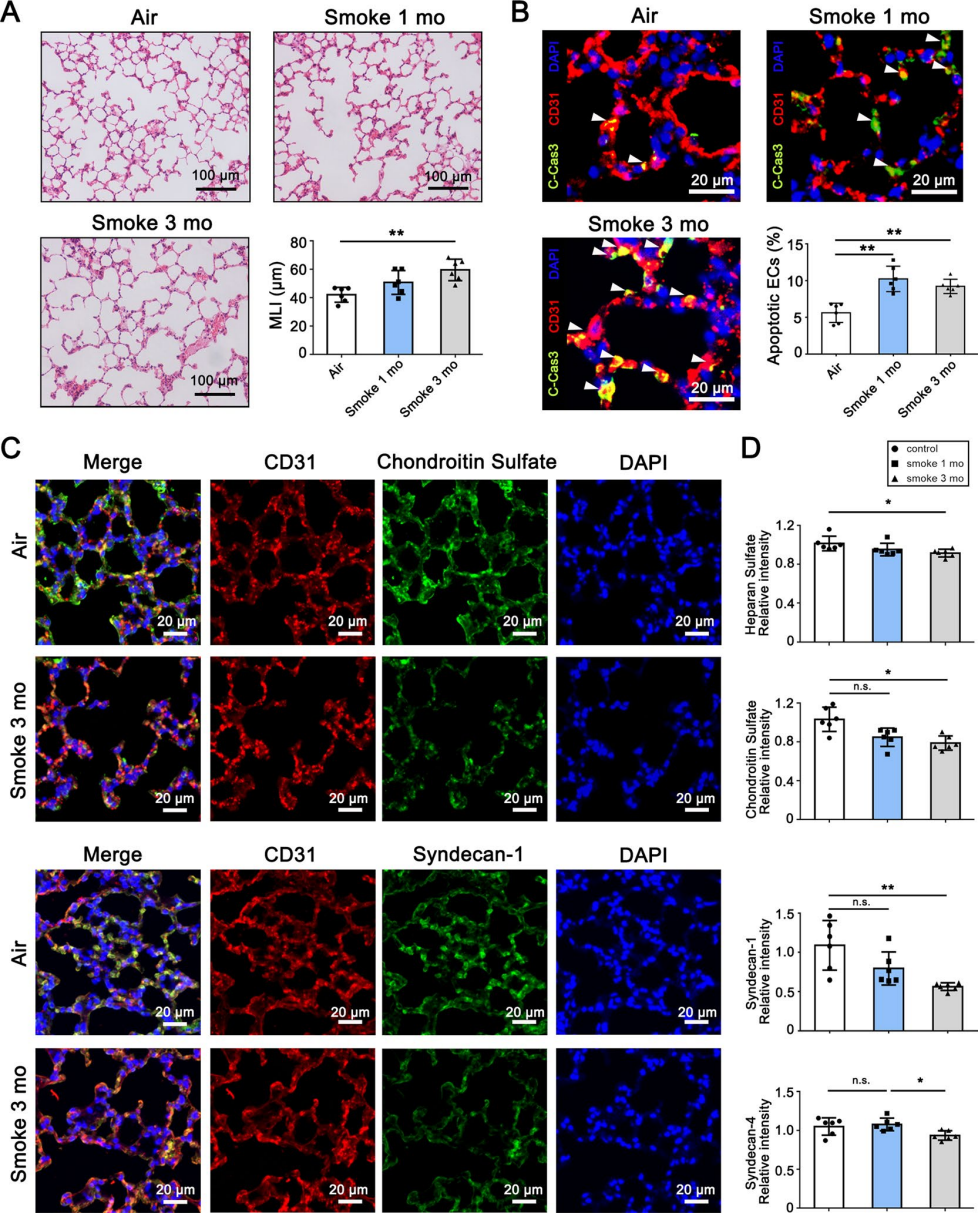
Exposure to cigarette smoke causes endothelial apoptosis, glycocalyx impairment and emphysema in mouse. Mice were exposed to air or cigarette smoke for 1 month or 3 months. **A** Representative haematoxylin and eosin (H&E) staining images of airspace. Alveolar size was measured by mean linear intercept (MLI) (n = 6). Scale bar = 100 μm. **B** Representative immunofluorescence staining images of cleaved caspase-3 in mice lungs. Nuclei were visualized with 4ʹ,6-diamidino-2-phenylindole (DAPI). White arrowheads indicate the apoptotic endothelial cells. Quantitative analysis of percentage of apoptotic endothelial cells was performed (n = 6). Scale bar = 20 μm. **C** Representative immunofluorescence staining images of mouse lung sections. Chondroitin sulfate and syndecan-1 antibodies were used as glycocalyx specific antigens, and CD31 was used as the mark of endothelial cell. Nuclei were visualized with DAPI. Scale bar = 20 μm. **D** Quantitative analysis of fluorescence intensity for heparan sulfate, chondroitin sulfate, syndecan-1 and syndecan-4 (n = 6). *P < 0.05, **P < 0.01. n.s., not significant

To investigate whether cigarette smoke exposure causes endothelial apoptosis and glycocalyx impairment, we first established the cigarette smoke-induced mouse COPD model. Mice were exposed to cigarette smoke for 1 month or 3 months. As compared to controls, mice exposed to cigarette smoke developed marked emphysematous changes as indicated by an increase in mean linear intercept (MLI) (Fig. 1A) and endothelial apoptosis (Fig. 1B). These effects were associated with the accumulation of endothelial glycocalyx impairment, evident as a decrease in endothelial glycocalyx components of lungs (Fig. 1C and D).

**Fig. 2:**
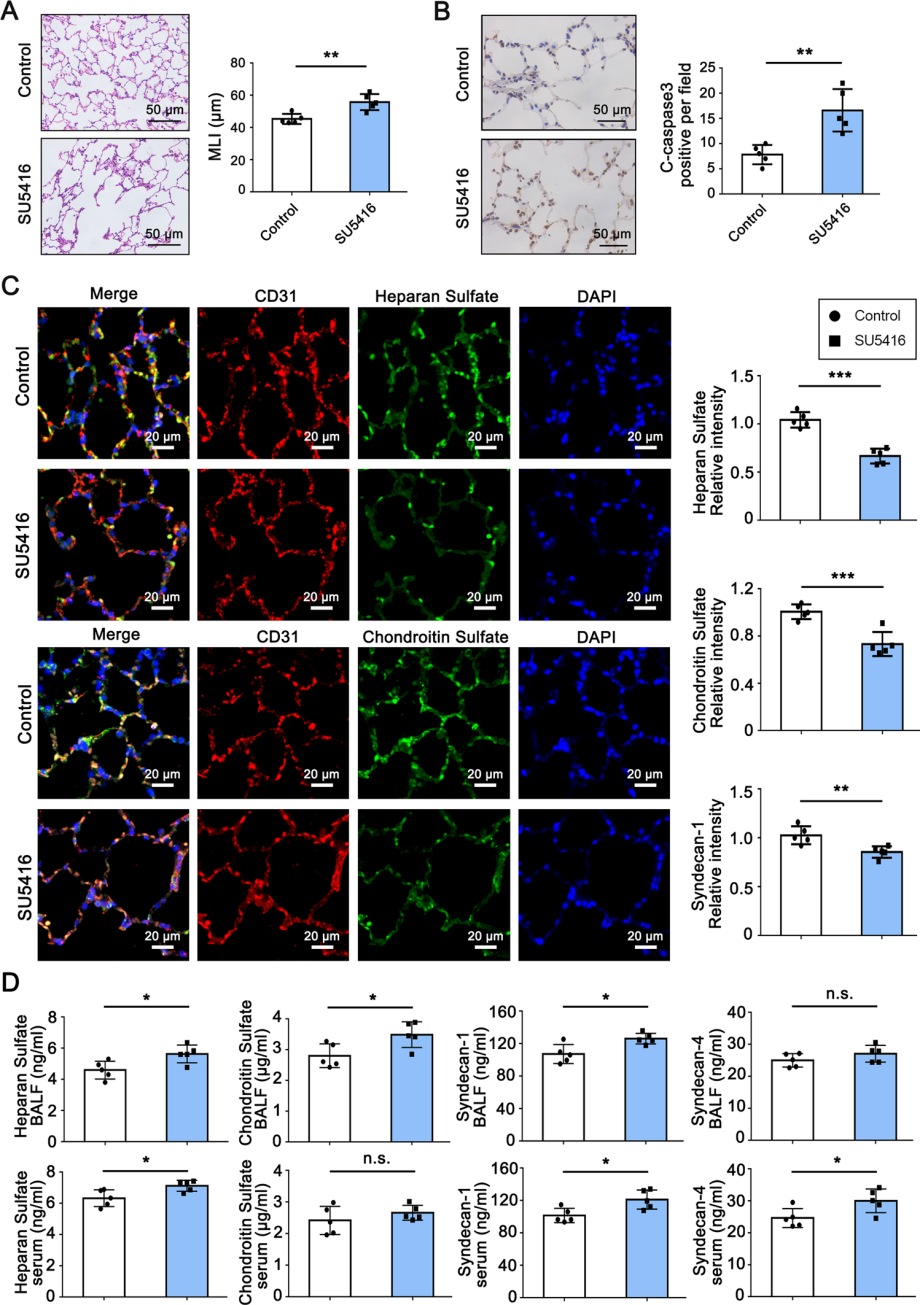
Endothelial glycocalyx impairment is involved in SU5416-induced rat emphysema model. Male Sprague-Dawley rats (~ 300 g) were treated with VEGF-R antagonist SU5416 (20 mg/kg) subcutaneously 3 times per week for 3 weeks. **A** Representative haematoxylin and eosin (H&E) staining images of airspace. Scale bar = 50 μm. Alveolar size was measured by mean linear intercept (MLI) (n = 5). **B** Cleaved caspase-3 antibody was used as the mark of cell apoptosis. Group data of cleaved caspase-3 positive cells per field (n = 5). Scale bar = 50 μm. **C** Representative immunofluorescence staining images of rat lung sections. Heparan sulfate and Chondroitin sulfate antibodies were used as glycocalyx specific antigens, and CD31 was used as the mark of endothelial cell. Nuclei were visualized with 4ʹ,6-diamidino-2-phenylindole (DAPI). Scale bar = 20 μm. Quantitative analysis of fluorescence intensity for heparan sulfate, chondroitin sulfate and syndecan-1 (n = 5). **D** ELISA analysis of blood and BALF samples (n = 5). *P < 0.05, **P < 0.01, ***P < 0.001. n.s., not significant

In order to test the specific role of endothelial cell apoptosis in glycocalyx injury and emphysema formation, we next treated rats with the synthetic VEGF-R antagonist SU5416, which blocks both VEGF-R_1_ and R_2_ [31]. SU5416 has been previously demonstrated to cause direct damage of endothelial cells and enlargement of the air spaces [3], a finding that could be replicated in the present study (Fig. 2A and B). Importantly, chronic treatment with SU5416 caused endothelial glycocalyx impairment, as illustrated by immunofluorescence images of decreased intensities of endothelial glycocalyx components (Fig. 2C), concomitantly with increased shedding of glycocalyx components both into circulation and into alveolar airspace (Fig. 2D), and mild dysfunction in alveolar-capillary barrier and lung permeability, as indicated by a mild but not significant increase in BALF total cell count and wet-to-dry ratio (Additional file 1: Figure S2). Together, these results suggest that the cigarette smoke-exposure induced endothelial glycocalyx impairment and emphysematous changes in animal models.

### Endothelial glycocalyx is impaired COPD patients

**Fig. 3:**
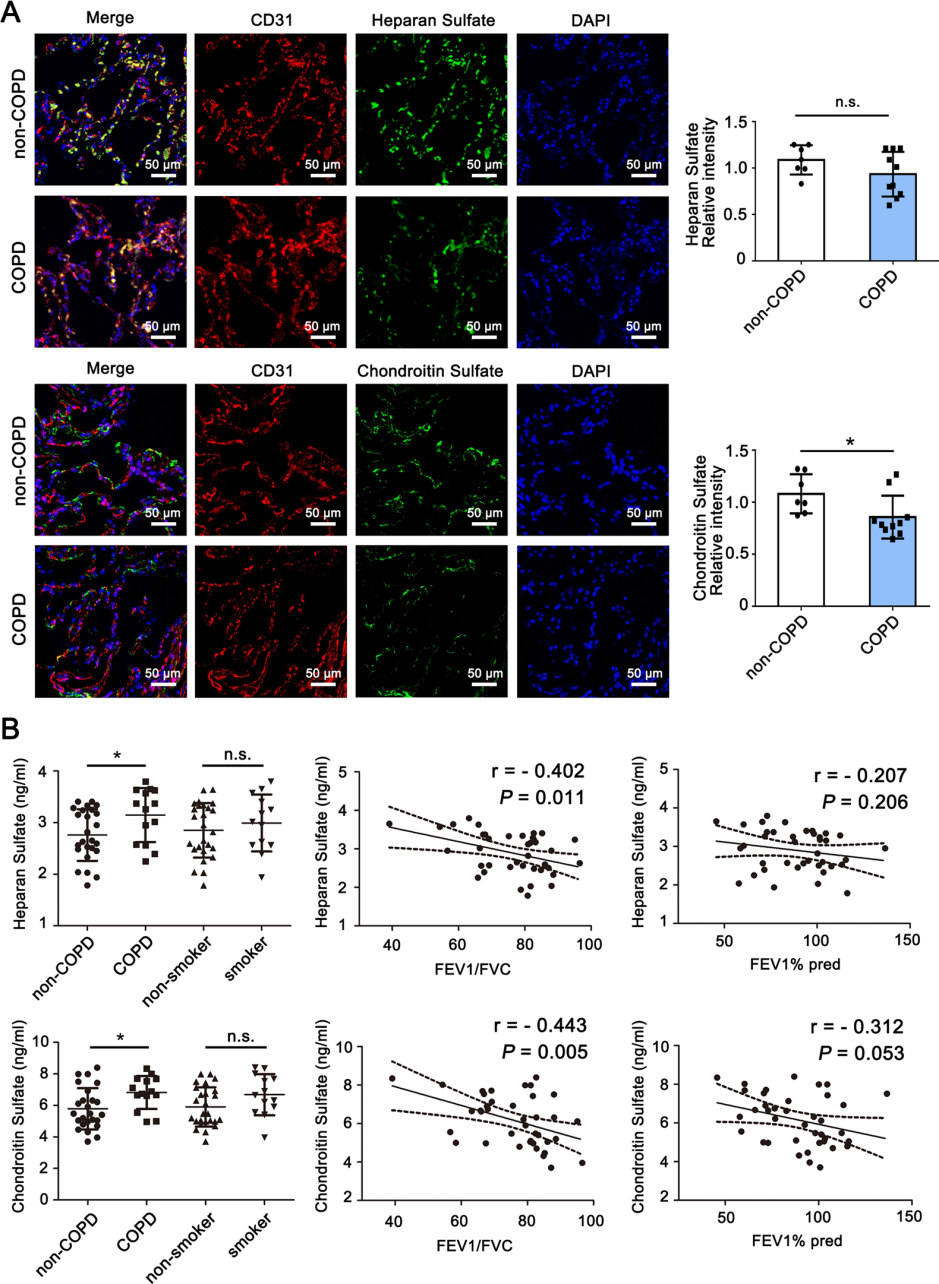
Endothelial glycocalyx is impaired in COPD patients. **A** Representative immunofluorescence staining images of heparan sulfate and chondroitin sulfate in non-COPD and COPD lungs. Heparan sulfate and chondroitin sulfate antibodies were used as glycocalyx specific antigens, and CD31 was used as the mark of endothelial cell. Nuclei were visualized with 4ʹ,6-diamidino-2-phenylindole (DAPI). Scale bar = 50 μm. Quantitative analysis of fluorescence intensity was performed (n = 7–10). **B** Serum heparan sulfate and chondroitin sulfate in subjects detected by ELISA analysis (n = 25 in non-COPD, n = 14 in COPD, n = 26 in non-smoker, n = 13 in smoker). Correlations of circulating heparan sulfate and chondroitin sulfate levels with lung function parameters (the ratio of FEV1/FVC and FEV1% predicted) in all subjects (n = 39) were analysed using Pearson correlation test. *P < 0.05. *n.s.*, not significant

To confirm the importance of endothelial glycocalyx in human COPD, we determined glycocalyx component expression in pulmonary endothelium from control subjects (non-COPD donors) or COPD patients applying immunofluorescence on human lung tissues. Non-COPD donors demonstrated diffuse expression of endothelial HS and CS, as demonstrated by HS and CS colocalization with endothelial marker CD31 (Fig. 3A). In contrast, COPD patients demonstrated a patchy loss of endothelial HS and CS expression. The serums of health donors (non-COPD) and patients with COPD were collected for measuring concentrations of glycocalyx components (Additional file 1: Table S1). In accordance with our findings in animal models, circulating concentrations of HS and CS were significantly elevated in patients with COPD, compared with non-COPD donors (Fig. 3B). Shedding of glycocalyx components in all subjects were positively correlated with declined lung function (the ratio of FEV1/FVC and FEV1% predicted) (Fig. 3B). However, we did not detect a trend toward syndecan-1 and syndecan-4 impairment in COPD patients (Additional file 1: Figure S3). Together, our clinical findings further suggest that endothelial glycocalyx is impaired in COPD.

### FGF10 attenuates the development of emphysema induced by cigarette smoke extract in mice

**Fig. 4:**
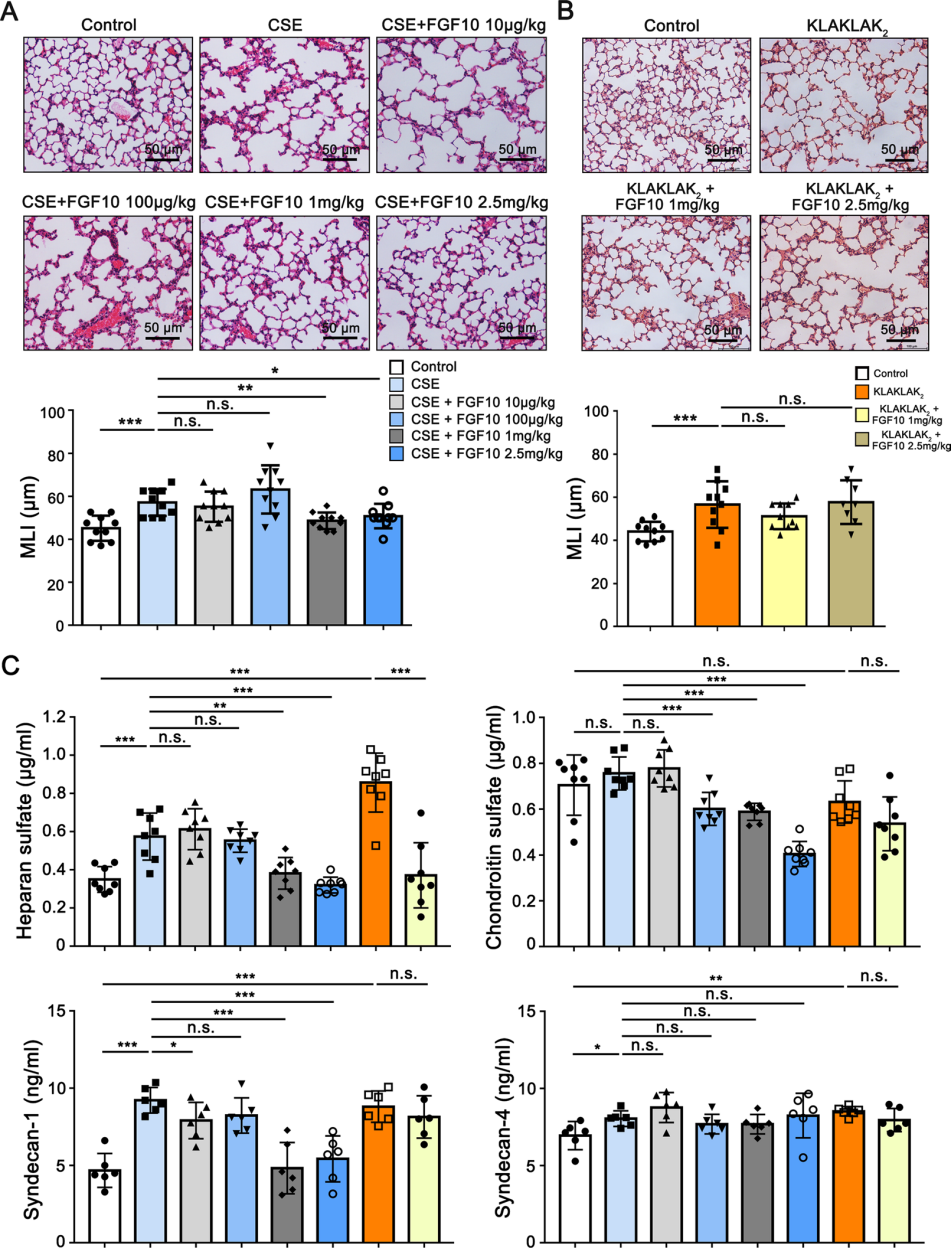
FGF10 attenuates the development of emphysema induced by cigarette smoke extract in mice. **A** Male C57BL/6 mice (6–8 weeks) were treated with 400 μl cigarette smoke extract (CSE) intraperitoneally once a week for 4 weeks, with different doses of FGF10 administration (10 μg/kg, 100 μg/kg, 1 mg/kg, 2.5 mg/kg) intravenously 24 h before CSE injection. All mice were sacrificed at Day 28. Representative haematoxylin and eosin (H&E) staining images of airspace. Scale bar = 50 μm. Alveolar size was evaluated by mean linear intercept (MLI) (n = 10). **B** Mice were administered standard saline solution or 240 μg of KLAKLAK_2_ intraperitoneally once per week for 4 weeks, with different doses of FGF10 administration intravenously 24 h prior to CSE injection. Representative H&E staining images of airspace. Scale bar = 50 μm. Alveolar size was evaluated by mean linear intercept (MLI), (n = 8–10). **C** Serum glycocalyx component levels in mice from each group detected by ELISA (n = 6–8). *P < 0.05, **P < 0.01, ***P < 0.001. n.s., not significant

To explore the protective effects of FGF10, cigarette smoke extract-exposed mice were challenged with recombinant human FGF10 in a dose-escalation manner. Lung histology assessment showed increased MLI in lungs of mice induced by CSE, and exogenous FGF10 attenuated emphysematous changes when the dose escalated to 1 mg/kg (Fig. 4A). In order to differentiate and probe for mechanistic signal pathways in endothelial cell apoptosis in COPD caused by either classical apoptosis inducer or cigarette smoking, we applied an endothelial-specific killing peptide KLAKLAK_2_ as positive controls (Additional file 1: Figure S4). The targeted induction of KLAKLAK_2_ caused emphysema-like changes in the mouse. However, 1 mg/kg or 2 mg/kg FGF10 did not alleviate the degree of emphysema (Fig. 4B). We measured circulating glycocalyx components of each group to determine whether FGF10 reduces endothelial glycocalyx shedding and impairment. The ELISA analysis showed that FGF10 attenuated HS and syndecan-1 shedding induced by CSE, but did not rescue the level of syndecan-4 (Fig. 4C).

**Fig. 5:**
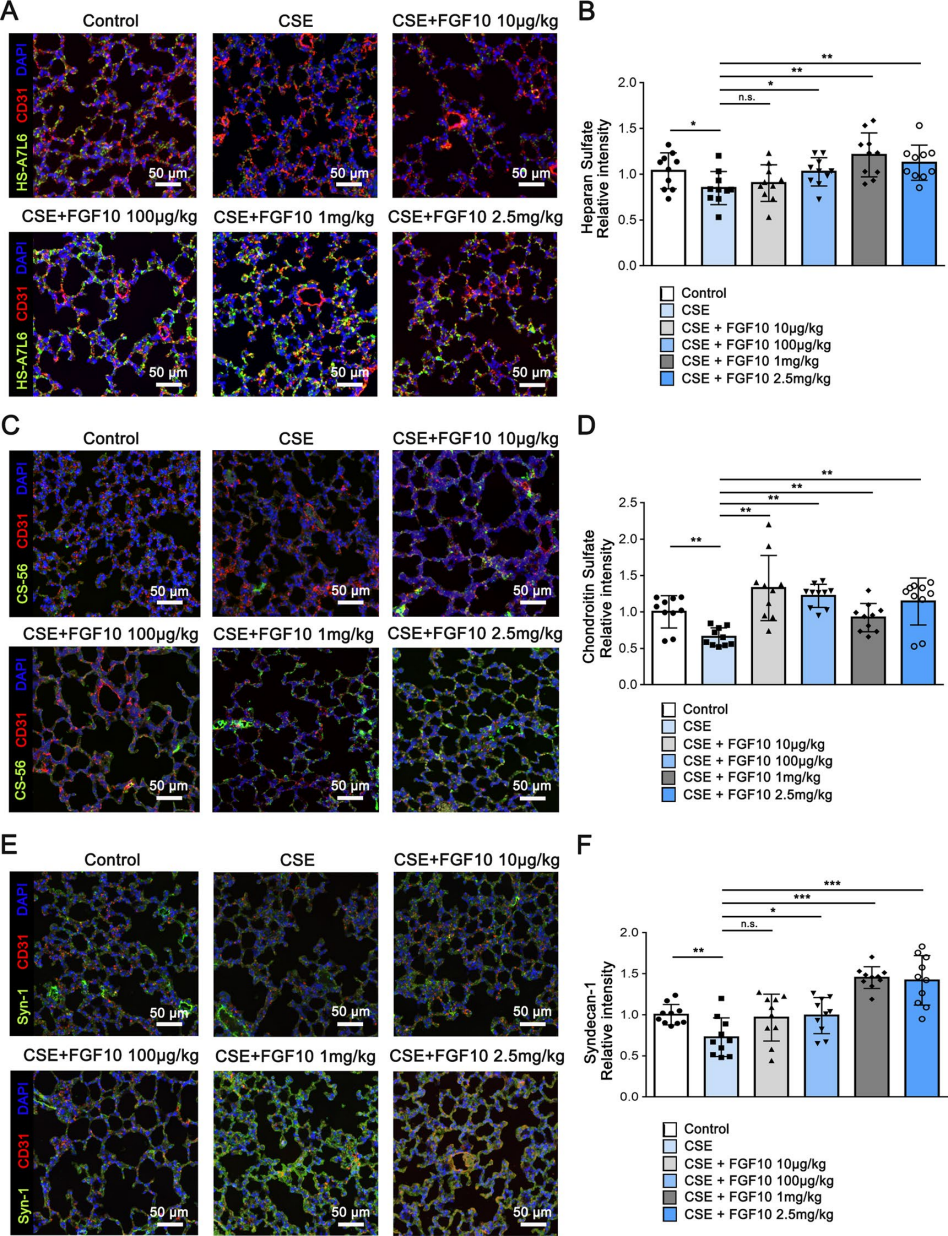
FGF10 increases the intensity of glycocalyx components in murine COPD model. Representative immunofluorescence staining images of heparan sulfate (**A**), chondroitin sulfate (**C**) and syndecan-1 (**E**) in mice lungs. Heparan sulfate, chondroitin sulfate and syndecan-1 antibodies were used as glycocalyx specific antigens, and CD31 was used as the mark of endothelial cell. Nuclei were visualized with 4ʹ,6-diamidino-2-phenylindole (DAPI). Scale bar = 50 μm. **B**, **D**, **F** Quantitative analysis of fluorescence intensity was performed (n = 10). *P < 0.05, **P < 0.01, ***P < 0.001. n.s., not significant

Parallelly, the glycocalyx components (HS, CS and syndecan-1) were stained with antibodies in mice lungs treated with CSE and FGF10 (Fig. 5). In line with previous results, CSE treatment caused endothelial glycocalyx impairment. Surprisingly, FGF10 increased the intensity of HS, CS and syndecan-1 even with a low dose of 10 or 100 μg/kg (Fig. 5), while FGF10 attenuated emphysematous changes when the dose escalated to at least 1 mg/kg (Fig. 4A), indicating that glycocalyx repair is a process prior to repair of emphysema. The results are also consistent with the alternations of circulating HS, CS and syndecan-1 (Fig. 4C), which maybe partially due to reduced shedding or increased reconstitution induced by FGF10.

### FGF10 attenuates cigarette smoke-induced endothelial apoptosis and glycocalyx repair through FGFR1 signaling

**Fig. 6:**
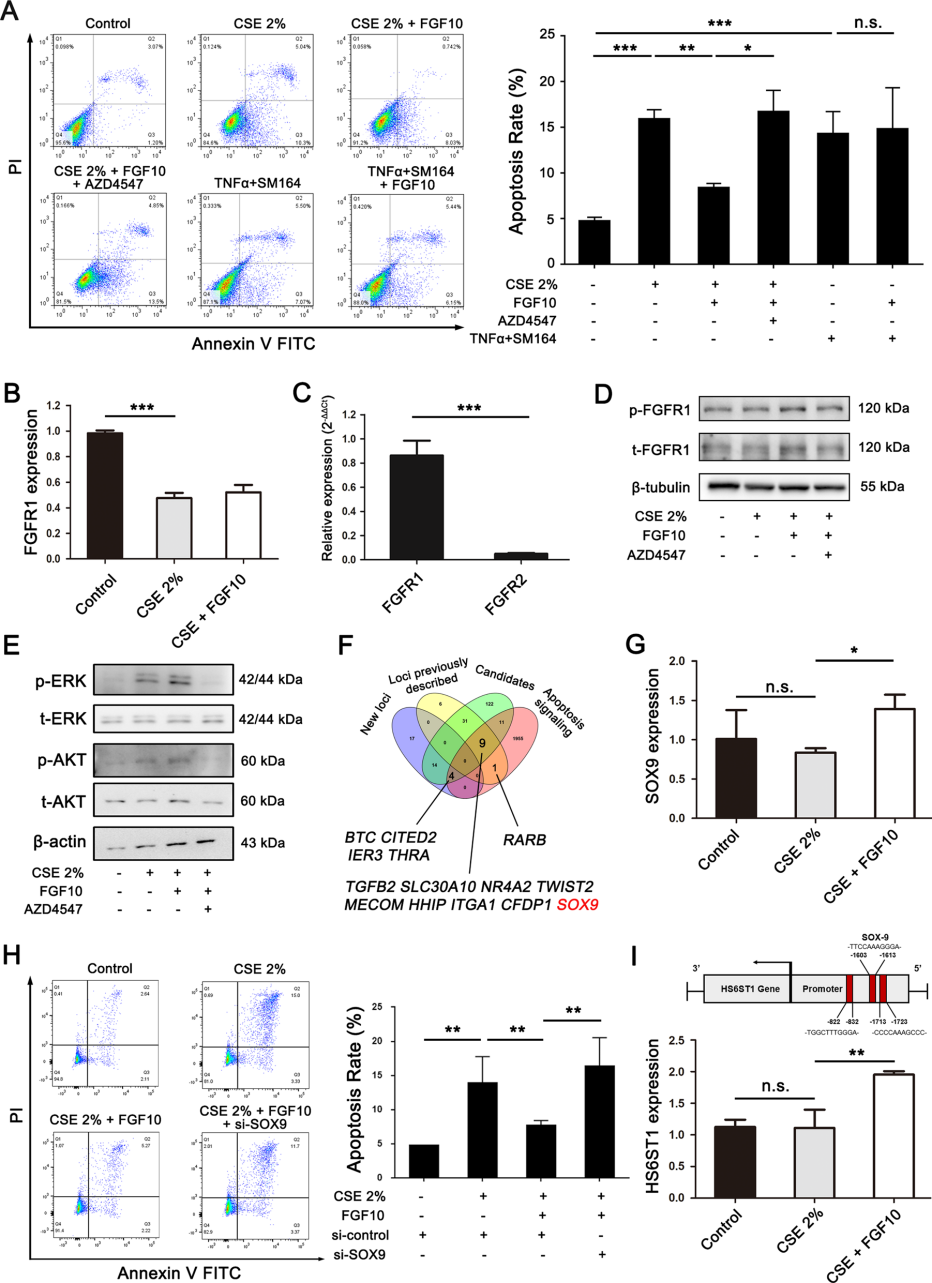
FGF10 attenuates cigarette smoke-induced endothelial apoptosis and glycocalyx repair through FGFR1 signaling. **A** The human pulmonary microvascular endothelial cells (hPMVECs) were treated with CSE (24 h, 2%), with or without pretreatment of FGF10 (2 h prior to CSE stimulation, 50 ng/ml) and the FGFR1 inhibitor, AZD4547 (24 h prior to CSE stimulation, 20 nM). TNF-α + SM-164 serves as positive controls (24 h, 1:500). After 24 h stimulation of 2% CSE, hPMVECs were performed for flow cytometry (n = 3). Annexin V/PI-stained cells showing apoptotic rates. FITC, fluorescein isothiocyanate; PI, propidium iodide. **B** Relative FGFR1 mRNA expression in hPMVECs from each group (n = 3). **C** Relative mRNA expression of FGFR1 and FGFR2 in hPMVECs (n = 3). **D** Representative western blots and corresponding group data showing expression levels of the total and phosphorylation of FGFR1. **E** Representative western blots and corresponding group data showing expression levels of the total and phosphorylation of ERK and AKT in hPMVECs. **F** Venn diagram of overlapping putative genes related both to cell apoptosis pathway and COPD, from “GO_Apoptosis_Signaling” (http://www.broadinstitute.org/gsea/index.jsp), and “New loci”, “Loci previously described” and “Candidates”. **G** Relative SOX9 mRNA expression in hPMVECs from each group (n = 3). **H** Endothelial cells were transfected with si-control and si-SOX9 for 48 h, stimulated beforehand with FGF10 (100 ng/ml) for 2 h, and then with CSE (2%) for another 24 h. Endothelial cells were performed for flow cytometry (n = 3). **I** Prediction of the combination site of SOX9 on the promoter region of HS6ST1. Relative HS6ST1 mRNA expression in hPMVECs from each group (n = 3). *P < 0.05, **P < 0.01, ***P < 0.001. n.s., not significant

To explore the potential molecular mechanisms of reparative FGF10, we conducted in vitro experiments using hPMVECs. The results showed that FGF10 attenuated 2% CSE-induced endothelial apoptosis (Fig. 6A). However, FGF10 did not reduce the EC apoptosis induced by TNFα + SM164, which served as a positive control, suggesting the specific protective role of FGF10 in CSE. Pretreatment with AZD4547, a high-affinity FGFR inhibitor, markedly impaired the protective effect of FGF10 on 2% CSE stimulation for hPMVECs, suggesting that FGF10 may attenuate CSE-induced COPD through FGFR signaling. Considering that only FGFR1 is suppressed during COPD (Fig. 6B and Additional file 1: Figure S5) and FGFR1 is the predominant FGFR expressed in pulmonary endothelial cells (Fig. 6C), we focused on the FGFR1 signaling, and found that FGF10 activated the FGFR1 and its downstream signaling ERK and AKT, which could be inhibited by AZD4547 (Fig. 6D and E). However, due to the lack of specific FGFR1 inhibitor, we still cannot exclude the compensatory role of FGFR2 in endothelial cells in response to smoking (Additional file 1: Figure S6), although FGFR2 expression is much lower than FGFR1 in ECs. Notably, our results showed that FGF10 treatment did not activate the FGFR3 signaling, and that the FGFR3 signaling was not inhibited by the AZD4547 with a concentration of 20 nM (Additional file 1: Figure S7).

**Fig. 7:**
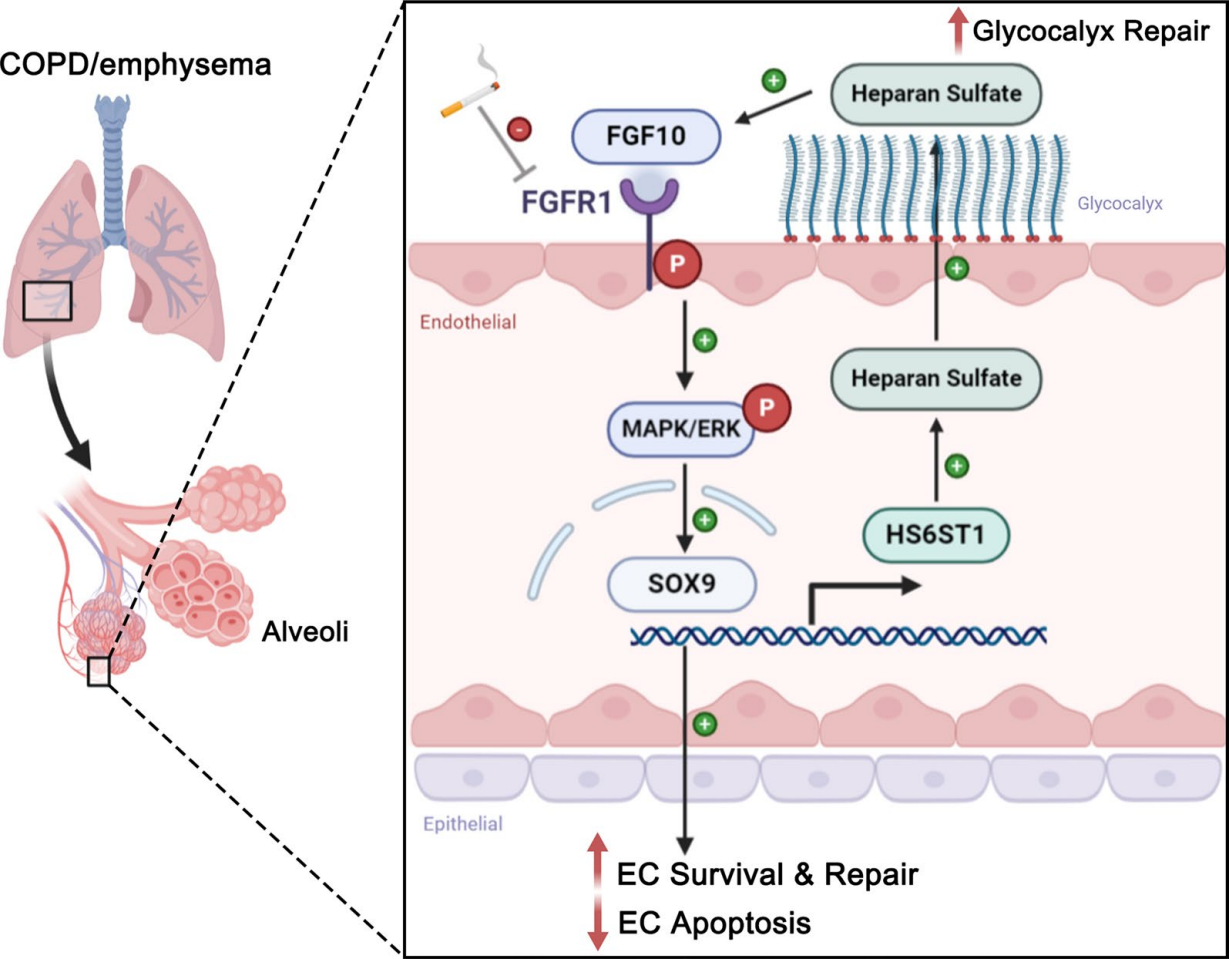
**Schematic concept of the FGF10/FGFR1/ERK/SOX9/HS6ST1/HS loop in glycocalyx synthesis and lung repair.** A model could be suggested in which cigarette smoke impairs endothelial glycocalyx and FGFR1 signaling, while FGF10 attenuates the development of emphysema and the shedding of glycocalyx induced by CSE in animal models. Mechanistically, FGF10 alleviates smoke-induced endothelial apoptosis and glycocalyx repair through a FGFR1/ERK/SOX9/HS6ST1 loop

Genetic risk loci provide new insights into COPD pathogenesis, especially for the genetic susceptibility and heterogeneity of disease. Sakornsakolpat et al. identified 82 loci in association with either COPD or population-based measures of lung function, including 47 previously described and 35 new [32]. To further explore potential mechanisms in CSE-induced EC apoptosis and glycocalyx repair, we screened overlapping genes related both to cell apoptosis pathway and COPD (Fig. 6F). Among 9 candidates from our screening result (*TGFB2*, *SLC30A10*, *NR4A2*, *TWIST2*, *MECOM*, *HHIP*, *ITGA1*, *CFDP1*, *SOX9*), Sex Determining Region Y-related HMG-box 9 (SOX9) was selected since: (1) SOX9 acts downstream of FGFR1/ERK signaling [33–35]; (2) SOX9 is essential for cell survival, and knockdown or dysfunction of SOX9 upregulates apoptosis-related genes [36, 37]; (3) SOX9 is highly related to COPD and is significantly associated with FEV1/FVC or FEV1 in meta-analysis models [32, 38, 39]; (4) *Sox9*−/− and *Sox9*+/− mice have severe tracheal cartilage malformations and die prematurely from respiratory insufficiency [40]; (5) SOX9 serves as a transcriptional factor, regulating the expression of heparan sulfate biosynthetic enzymes, which are required for the synthesis and function of glycocalyx/HS, leading to a SOX9/FGF feed-forward loop [33, 41]. Thus, we decided to focus on the characterization of SOX9 in CSE-induced cell apoptosis. Indeed, SOX9 mRNA level was significantly increased when hPMVECs were treated with FGF10 prior to CSE exposure, compared to CSE stimulation (Fig. 6G). We further demonstrated that knockdown of SOX9 could attenuate the protective effect of FGF10 on endothelial cell apoptosis (Additional file 1: Figure S8 and Fig. 6H). We also predicted the combination site of SOX9 on the promoter region of heparan sulfate 6-*O*-sulfotransferase 1 (HS6ST1) (− 2000 to 0 bp), which is a heparan sulfate biosynthetic enzyme. In parallel with SOX9, pretreatment with FGF10 also upregulated HS6ST1 expression (Fig. 6I), suggesting a potential FGF10/FGFR1/ERK/SOX9/HS6ST1/HS loop involved in glycocalyx synthesis and repair (Fig. 7).

## Discussion

In this study, we first proved the coexistence of endothelial apoptosis and glycocalyx impairment in COPD and its most important phenotype emphysema, using animal models and clinical samples. Interestingly, in the emphysema model induced by VEGF-R blockade, glycocalyx accumulation was decreased in alveoli and glycocalyx shedding was elevated both in circulation and alveolar space. These findings strongly reinforced the hypothesis whereby apoptosis is the primary event that leads to loss of alveolar units and emphysema [3, 5, 42]. Importantly, administration of FGF10 rescued glycocalyx impairment and emphysema in a murine model of COPD, probably through a FGFR1/ERK/SOX9/HS6ST1 loop in endothelial cells.

FGF10 presents an attractive candidate in the protection and repair of COPD as (1) FGF10 is dysregulated in various human lung diseases including COPD, and (2) FGF10 haploinsufficiency affects lung function that may ultimately lead to the development of COPD [20–22]. Here, we demonstrated that intravenous injection of FGF10 has a protective effect on CSE-induced glycocalyx impairment and emphysema in a dose-dependent manner. In contrast, FGF10 with a dose of 1 or 2 mg/kg did not prevent KLAKLAK_2_-induced emphysema which serves as positive controls. We thought it might be due to different pathogenic mechanisms between CSE and KLAKLAK_2_. While CSE may disrupt the FGFR1 signaling in endothelial cells, KLAKLAK_2_ is a chimera lung homing peptide specific for lung endothelial cells, which disrupts anionic phospholipids present in mitochondria, leading to apoptotic endothelial cell death [30]. Considering the protective effects of FGF10 on epithelial injury induced by multiple mediators, FGF10 was injected intravenously instead of intratracheally in our animal study, to focus on the protective role of FGF10 on endothelium. To explore the role of FGF10 on epithelial cells, we additionally cultured two types of epithelial cells BEAS-2B and 16HBE, and treated them with FGF10. Our results showed that FGF10 treatment did not activate the FGFR1 signaling and its downstream AKT, but could activate the FGFR2/3 in epithelium (Additional file 1: Figures S9 and S10). It was proposed that FGF10 acted on endothelial cells differently from epithelial cells which through their typical receptors.

The cellular mechanism that mediates endothelial survival and glycocalyx repair depends on FGFR1 signaling [10]. In line with previous reports, our findings suggest that FGF10 inhibits the CSE-induced endothelial apoptosis mainly through FGFR1 signaling as (1) database analysis revealed that FGFR1, but not FGFR2, is suppressed during COPD, (2) FGFR1, but not FGFR2, is the predominant FGFR expressed in pulmonary endothelial cells, and (3) FGF10 serves as a ligand binding to FGFR1b, though with a relative low affinity compared to FGFR2b [19, 43]. However, the compensatory role of FGFR2 in endothelial cells still cannot be excluded in response to smoking.

SOX9 was identified using bioinformatic analysis to explore potential mechanisms in CSE-induced EC apoptosis and glycocalyx repair. SOX9 acts downstream of FGFR1/ERK pathway [33–35] and relates to both cell apoptosis [36, 37] and COPD [32, 38, 39]. In turn, SOX9 regulates the expression of heparan sulfate biosynthetic enzyme HS6ST1, which is required for synthesis and function of heparan sulfate [33, 41]. HS6ST1 is considered to be essential in lung development, and *HS6ST1*-deficiency in mice exhibit airspace enlargement during postnatal period associated with abnormal elastin deposition [44]. In this study, pretreatment with FGF10 significantly increased the SOX9 mRNA expression in endothelial cells treated with CSE, and FGF10 up-regulated the expression of HS6ST1 in parallel with SOX9. We further demonstrated that knockdown of SOX9 could attenuate the protective effect of FGF10 on endothelial cell apoptosis, suggesting a FGF10/FGFR1/ERK/SOX9/HS6ST1/HS loop involved in glycocalyx repair and endothelial apoptosis.

When anchored to proteoglycans on cell surface, HS may function as an activating coreceptor for growth factor ligand–receptor interaction [45]. The biological activities of FGF10 can be modulated by HS structures with specific sulfate patterns and density [46]. Yang’s work highlights the importance of endothelial surface layer (ESL)-derived HS fragments in promoting FGFR1 signaling. HS degradation of sufficient size and appropriate N-sulfation during sepsis can bind to FGF2, activating and augmenting FGF2-FGFR1 signaling [10]. In contrast, LaRivière and colleagues suggest that heparan sulfate shed into the airspace after injury may directly impair lung repair, by binding to hepatocyte growth factor (HGF) and attenuates growth factor signaling [47]. Therefore, determination of HS or other glycocalyx shedding and patterns of interaction with FGF10 during COPD, is required in the future. Integrating the HS into our picture of the FGF10 also provides attractive therapeutic perspectives by adding HS to exogenous FGF10 preparations.

Some limitations should be clarified. First, we used three different animal models of COPD and emphysema. Animal model of smoke-induced COPD is the primary testing methodology for drug therapies and studies on pathogenic mechanisms of COPD/emphysema [48]. Apart from this classic model, VEGF-R antagonist SU5416 was also used in this study to investigate the specific role of endothelial apoptosis in emphysema formation. To evaluate the protective effect of FGF10, we did not apply cigarette smoke exposure models, but used intraperitoneal administration of CSE instead for the following reasons: (1) the pathological emphysematous phenotype rather than an inflammatory phenotype is more viable induced by sterile CSE injection intraperitoneally, compared to whole-body or nose cigarette smoking exposure [26, 49]; (2) cigarette smoking exposure directly targets epithelial cells, while cigarette smoke extract injected intraperitoneally directly targets endothelial cells; and (3) endothelial-specific killing peptide served as a positive control is also injected intraperitoneally [30]. Second, our study suggests that cigarette smoke exposure caused the loss of FGFR1 expression and impairment of FGFR1 signaling, and that FGF10 attenuates cigarette smoke-induced COPD through FGFR1 signaling; further confirmatory studies, including the use of *FGFR1* knockout mice, are necessary to fully elucidate the precise associated mechanisms. Third, cigarette smoking exposure could cause excessive endothelial apoptosis and emphysema through other signaling pathways involving downregulation of vascular endothelial growth factor (VEGF), focal adhesion kinase (FAK), α-1-antitrypsin (AAT), and upregulation of ceramide, p38, and p53 [50]. FGF10 was reported to effectively inhibit apoptosis and maintain endothelial barrier function through phosphoinositide 3-kinase (PI3K) or epidermal growth factor receptor (EGFR) signal pathways [28]. Therefore, further studies are needed to clarify the crosstalk between these receptors, and to explore whether FGF10 attenuates endothelial apoptosis and emphysema through pathways other than FGFR1.

## Conclusions

FGF10 treatment was shown to repair pulmonary glycocalyx injury and endothelial apoptosis, and attenuate smoke-induced COPD through FGFR1 signaling. Since FGF10 is currently used in the clinical setting, it may serve as a potential therapeutic strategy against COPD via endothelial repair and glycocalyx reconstitution.

## Supplementary Information

**Additional file 1.** Supplementary figures and tables.

## Data Availability

The data used in this study are available from the corresponding authors on reasonable request.

## References

[1] Collaborators GBDCRD (2017). Global, regional, and national deaths, prevalence, disability-adjusted life years, and years lived with disability for chronic obstructive pulmonary disease and asthma, 1990–2015: a systematic analysis for the Global Burden of Disease Study 2015. Lancet Respir Med.

[2] Chandra D, Sciurba FC, Gladwin MT (2011). Endothelial chronic destructive pulmonary disease (E-CDPD): is endothelial apoptosis a subphenotype or prequel to COPD?. Am J Respir Crit Care Med.

[3] Kasahara Y, Tuder RM, Taraseviciene-Stewart L, Le Cras TD, Abman S, Hirth PK, Waltenberger J, Voelkel NF (2000). Inhibition of VEGF receptors causes lung cell apoptosis and emphysema. J Clin Invest.

[4] Haeger SM, Liu X, Han X, McNeil JB, Oshima K, McMurtry SA, Yang Y, Ouyang Y, Zhang F, Nozik-Grayck E (2018). Epithelial heparan sulfate contributes to alveolar barrier function and is shed during lung injury. Am J Respir Cell Mol Biol.

[5] Aoshiba K, Yokohori N, Nagai A (2003). Alveolar wall apoptosis causes lung destruction and emphysematous changes. Am J Respir Cell Mol Biol.

[6] Agusti A, Hogg JC (2019). Update on the pathogenesis of chronic obstructive pulmonary disease. N Engl J Med.

[7] Weidenfeld S, Kuebler WM (2018). Shedding first light on the alveolar epithelial glycocalyx. Am J Respir Cell Mol Biol.

[8] Reitsma S, Slaaf DW, Vink H, van Zandvoort MA, Oude Egbrink MG (2007). The endothelial glycocalyx: composition, functions, and visualization. Pflugers Arch.

[9] Alphonsus CS, Rodseth RN (2014). The endothelial glycocalyx: a review of the vascular barrier. Anaesthesia.

[10] Yang Y, Haeger SM, Suflita MA, Zhang F, Dailey KL, Colbert JF, Ford JA, Picon MA, Stearman RS, Lin L (2017). Fibroblast growth factor signaling mediates pulmonary endothelial glycocalyx reconstitution. Am J Respir Cell Mol Biol.

[11] Potter DR, Jiang J, Damiano ER (2009). The recovery time course of the endothelial cell glycocalyx in vivo and its implications in vitro. Circ Res.

[12] Rizzo AN, Dudek SM (2017). Endothelial glycocalyx repair: building a wall to protect the lung during sepsis. Am J Respir Cell Mol Biol.

[13] Ornitz DM, Itoh N (2015). The fibroblast growth factor signaling pathway. Wiley Interdiscip Rev Dev Biol.

[14] El Agha E, Schwind F, Ruppert C, Gunther A, Bellusci S, Schermuly RT, Kosanovic D (2018). Is the fibroblast growth factor signaling pathway a victim of receptor tyrosine kinase inhibition in pulmonary parenchymal and vascular remodeling?. Am J Physiol Lung Cell Mol Physiol.

[15] Kranenburg AR, De Boer WI, Van Krieken JH, Mooi WJ, Walters JE, Saxena PR, Sterk PJ, Sharma HS (2002). Enhanced expression of fibroblast growth factors and receptor FGFR-1 during vascular remodeling in chronic obstructive pulmonary disease. Am J Respir Cell Mol Biol.

[16] Itoh N (2016). FGF10: a multifunctional mesenchymal-epithelial signaling growth factor in development, health, and disease. Cytokine Growth Factor Rev.

[17] Sekine K, Ohuchi H, Fujiwara M, Yamasaki M, Yoshizawa T, Sato T, Yagishita N, Matsui D, Koga Y, Itoh N, Kato S (1999). Fgf10 is essential for limb and lung formation. Nat Genet.

[18] Min H, Danilenko DM, Scully SA, Bolon B, Ring BD, Tarpley JE, DeRose M, Simonet WS (1998). Fgf-10 is required for both limb and lung development and exhibits striking functional similarity to Drosophila branchless. Genes Dev.

[19] Ohuchi H, Hori Y, Yamasaki M, Harada H, Sekine K, Kato S, Itoh N (2000). FGF10 acts as a major ligand for FGF receptor 2 IIIb in mouse multi-organ development. Biochem Biophys Res Commun.

[20] Finch PW, Mark Cross LJ, McAuley DF, Farrell CL (2013). Palifermin for the protection and regeneration of epithelial tissues following injury: new findings in basic research and pre-clinical models. J Cell Mol Med.

[21] Yuan T, Volckaert T, Chanda D, Thannickal VJ, De Langhe SP (2018). Fgf10 signaling in lung development, homeostasis, disease, and repair after injury. Front Genet.

[22] Klar J, Blomstrand P, Brunmark C, Badhai J, Hakansson HF, Brange CS, Bergendal B, Dahl N (2011). Fibroblast growth factor 10 haploinsufficiency causes chronic obstructive pulmonary disease. J Med Genet.

[23] Zhang LQ, Cheranova D, Gibson M, Ding S, Heruth DP, Fang D, Ye SQ (2012). RNA-seq reveals novel transcriptome of genes and their isoforms in human pulmonary microvascular endothelial cells treated with thrombin. PLoS ONE.

[24] Graham BL, Steenbruggen I, Miller MR, Barjaktarevic IZ, Cooper BG, Hall GL, Hallstrand TS, Kaminsky DA, McCarthy K, McCormack MC (2019). Standardization of Spirometry 2019 Update. An official American Thoracic Society and European Respiratory Society Technical Statement. Am J Respir Crit Care Med.

[25] Vogelmeier CF, Criner GJ, Martinez FJ, Anzueto A, Barnes PJ, Bourbeau J, Celli BR, Chen R, Decramer M, Fabbri LM (2017). Global strategy for the diagnosis, management, and prevention of chronic obstructive lung disease 2017 report. GOLD executive summary. Am J Respir Crit Care Med.

[26] Morichika D, Miyahara N, Fujii U, Taniguchi A, Oda N, Senoo S, Kataoka M, Tanimoto M, Kakuta H, Kiura K (2019). A retinoid X receptor partial agonist attenuates pulmonary emphysema and airway inflammation. Respir Res.

[27] Bi J, Tong L, Zhu X, Yang D, Bai C, Song Y, She J (2014). Keratinocyte growth factor-2 intratracheal instillation significantly attenuates ventilator-induced lung injury in rats. J Cell Mol Med.

[28] Fang X, Wang L, Shi L, Chen C, Wang Q, Bai C, Wang X (2014). Protective effects of keratinocyte growth factor-2 on ischemia-reperfusion-induced lung injury in rats. Am J Respir Cell Mol Biol.

[29] She J, Goolaerts A, Shen J, Bi J, Tong L, Gao L, Song Y, Bai C (2012). KGF-2 targets alveolar epithelia and capillary endothelia to reduce high altitude pulmonary oedema in rats. J Cell Mol Med.

[30] Giordano RJ, Lahdenranta J, Zhen L, Chukwueke U, Petrache I, Langley RR, Fidler IJ, Pasqualini R, Tuder RM, Arap W (2008). Targeted induction of lung endothelial cell apoptosis causes emphysema-like changes in the mouse. J Biol Chem.

[31] Fong TA, Shawver LK, Sun L, Tang C, App H, Powell TJ, Kim YH, Schreck R, Wang X, Risau W (1999). SU5416 is a potent and selective inhibitor of the vascular endothelial growth factor receptor (Flk-1/KDR) that inhibits tyrosine kinase catalysis, tumor vascularization, and growth of multiple tumor types. Cancer Res.

[32] Sakornsakolpat P, Prokopenko D, Lamontagne M, Reeve NF, Guyatt AL, Jackson VE, Shrine N, Qiao D, Bartz TM, Kim DK (2019). Genetic landscape of chronic obstructive pulmonary disease identifies heterogeneous cell-type and phenotype associations. Nat Genet.

[33] Chen Z, Huang J, Liu Y, Dattilo LK, Huh SH, Ornitz D, Beebe DC (2014). FGF signaling activates a Sox9-Sox10 pathway for the formation and branching morphogenesis of mouse ocular glands. Development.

[34] Murakami S, Kan M, McKeehan WL, de Crombrugghe B (2000). Up-regulation of the chondrogenic Sox9 gene by fibroblast growth factors is mediated by the mitogen-activated protein kinase pathway. Proc Natl Acad Sci USA.

[35] Acevedo VD, Gangula RD, Freeman KW, Li R, Zhang Y, Wang F, Ayala GE, Peterson LE, Ittmann M, Spencer DM (2007). Inducible FGFR-1 activation leads to irreversible prostate adenocarcinoma and an epithelial-to-mesenchymal transition. Cancer Cell.

[36] Jiang SS, Fang WT, Hou YH, Huang SF, Yen BL, Chang JL, Li SM, Liu HP, Liu YL, Huang CT (2010). Upregulation of SOX9 in lung adenocarcinoma and its involvement in the regulation of cell growth and tumorigenicity. Clin Cancer Res.

[37] Ma Y, Shepherd J, Zhao D, Bollu LR, Tahaney WM, Hill J, Zhang Y, Mazumdar A, Brown PH (2020). SOX9 is essential for triple-negative breast cancer cell survival and metastasis. Mol Cancer Res.

[38] Hancock DB, Soler Artigas M, Gharib SA, Henry A, Manichaikul A, Ramasamy A, Loth DW, Imboden M, Koch B, McArdle WL (2012). Genome-wide joint meta-analysis of SNP and SNP-by-smoking interaction identifies novel loci for pulmonary function. PLoS Genet.

[39] Wang R, Ahmed J, Wang G, Hassan I, Strulovici-Barel Y, Hackett NR, Crystal RG (2011). Down-regulation of the canonical Wnt beta-catenin pathway in the airway epithelium of healthy smokers and smokers with COPD. PLoS ONE.

[40] Bi W, Huang W, Whitworth DJ, Deng JM, Zhang Z, Behringer RR, de Crombrugghe B (2001). Haploinsufficiency of Sox9 results in defective cartilage primordia and premature skeletal mineralization. Proc Natl Acad Sci USA.

[41] Seymour PA, Shih HP, Patel NA, Freude KK, Xie R, Lim CJ, Sander M (2012). A Sox9/Fgf feed-forward loop maintains pancreatic organ identity. Development.

[42] Tuder RM, Petrache I, Elias JA, Voelkel NF, Henson PM (2003). Apoptosis and emphysema: the missing link. Am J Respir Cell Mol Biol.

[43] Emoto H, Tagashira S, Mattei MG, Yamasaki M, Hashimoto G, Katsumata T, Negoro T, Nakatsuka M, Birnbaum D, Coulier F, Itoh N (1997). Structure and expression of human fibroblast growth factor-10. J Biol Chem.

[44] Habuchi H, Nagai N, Sugaya N, Atsumi F, Stevens RL, Kimata K (2007). Mice deficient in heparan sulfate 6-O-sulfotransferase-1 exhibit defective heparan sulfate biosynthesis, abnormal placentation, and late embryonic lethality. J Biol Chem.

[45] Goetz R, Mohammadi M (2013). Exploring mechanisms of FGF signalling through the lens of structural biology. Nat Rev Mol Cell Biol.

[46] Patel VN, Likar KM, Zisman-Rozen S, Cowherd SN, Lassiter KS, Sher I, Yates EA, Turnbull JE, Ron D, Hoffman MP (2008). Specific heparan sulfate structures modulate FGF10-mediated submandibular gland epithelial morphogenesis and differentiation. J Biol Chem.

[47] LaRiviere WB, Liao S, McMurtry SA, Oshima K, Han X, Zhang F, Yan S, Haeger SM, Ransom M, Bastarache JA (2020). Alveolar heparan sulfate shedding impedes recovery from bleomycin-induced lung injury. Am J Physiol Lung Cell Mol Physiol.

[48] Shu J, Li D, Ouyang H, Huang J, Long Z, Liang Z, Chen Y, Chen Y, Zheng Q, Kuang M (2017). Comparison and evaluation of two different methods to establish the cigarette smoke exposure mouse model of COPD. Sci Rep.

[49] Hanaoka M, Droma Y, Chen Y, Agatsuma T, Kitaguchi Y, Voelkel NF, Kubo K (2011). Carbocisteine protects against emphysema induced by cigarette smoke extract in rats. Chest.

[50] Lu Q, Gottlieb E, Rounds S (2018). Effects of cigarette smoke on pulmonary endothelial cells. Am J Physiol Lung Cell Mol Physiol.

