# Supplementary material for: Fibroblast growth factor 10 attenuates chronic obstructive pulmonary disease by protecting against glycocalyx impairment and endothelial apoptosis
Source: Respir Res. 2022 Oct 1;23:269. doi: 10.1186/s12931-022-02193-5 (PMC9526324; doi:10.1186/s12931-022-02193-5)
Supplement: Supplementary file 1 — Additional file 1. Supplementary figures and tables. [file 12931_2022_2193_MOESM1_ESM.doc]

**Fibroblast growth factor 10 attenuates chronic obstructive pulmonary disease by protecting against glycocalyx impairment and endothelial apoptosis**

Tian Jiang, Weiping Hu, Shaoyuan Zhang, Changhao Ren, Siyun Lin, Zhenyu Zhou, Hao Wu, Jun Yin, Lijie Tan

***Supplementary Materials***

***Detailed Methods***

***Cigarette smoke extract***

Cigarette smoke extract (CSE) was prepared as previously described [1]. The smoke from five cigarettes, with each containing 11 mg tar and 0.8 mg nicotine (Daqianmen, China), was bubbled through 10 ml of 1640 culture medium (Gibco, Thermo Fisher Scientific, Inc., Waltham, MA, USA). The CSE filtrate was then passed through a 0.22 µm pore size filter (Millipore, Billerica, MA, USA) to remove particles and bacteria. This product was defined as 100% CSE. The half maximal inhibitory concentration (IC50) of CSE to endothelial cells was ~2% (**Supplementary Figure 1**). For sterile CSE used for mouse emphysema model (intraperitoneally), the smoke from one cigarette was bubbled through 10 ml of PBS (Gibco), and then filtered as previously described.

***Cell culture***

Human pulmonary microvascular endothelial cells (hPMVECs, passages 4-6, Cat #C-12281, PromoCell, Heidelberg, Germany) were used in this study. Cells were cultured with Endothelial Cell Growth Medium MV2 (Cat #C-22022, PromoCell) at 37 °C in a humidified atmosphere containing 5% CO_2_. The hPMVECs were treated with different concentrations of CSE, 50 ng/ml human recombinant FGF10 (R&D Systems, Minneapolis, MN) at the presence or absence of 20 nM FGFR1 inhibitor AZD4547 (MedChemExpress, Monmouth Junction, NJ, USA). The dose of FGF10 for hPMVECs was selected based on previous results [2]. The dose of AZD4547 was selected based on previous report [3] and our preliminary data showing that viability of hPMVECs will not be obviously affected until the concentration of AZD4547 reached at over 50 nM.

***Animal samples processing***

Serum was collected and isolated using serum separator tubes (Becton Dickinson). When we processed the rat lungs, we blocked the right main bronchus by a clip, and collected the bronchoalveolar lavage fluid (BALF) from the left lungs with three – one milliliter cold PBS for further analysis. The right upper lobes of lungs were collected for morphologic analysis (H&E), and the remaining lungs (right middle and lower lobes) were inflated manually with PBS/OCT (50%/50%) with subsequent snap-frozen in liquid nitrogen. For the mice, all lungs were fixed in 10% formalin and paraffin-embedded for further IF or IHC assays.

***Morphologic Analysis***

The paraffin-embedded tissue sections (4 μm-thick) were stained with hematoxylin and eosin (H&E). Mean linear intercept (MLI) was measured by Image J software, as described previously [4]. For each sample, five random fields were analyzed. From each field, 10 areas of interest, free of airways and blood vessels, were picked for measurement of the number of intersections of virtual lines of known length, with alveolar septa. The MLI was calculated by dividing the line length by the total number of intercepts. An increase in the average distance between intercepts (MLI) indicates enlarged airspaces.

***Immunofluorescence (IF)***

Immunofluorescence was performed on 7-μm sections from paraffin-embedded or frozen animal lungs. The antibodies used in this study were summarized in **Supplementary Table 2**. Primary antibodies included anti-Syndecan-1 antibody (Abcam, Cambridge, MA, USA), anti-Syndecan-4 antibody (Abcam), anti-Heparan Sulfate antibody (Abcam), anti-Chondroitin Sulfate antibody (Abcam) and anti-CD31 antibody (Abcam), followed by Alexa Fluor 488 or 546 as the second antibody. The DyLight 594 labeled Lycopersicon Esculentum (Tomato) Lectin (Vector; Burlingame, CA, USA) was used to indicate the endothelial cells or vascular structures. Nuclei were visualized with 4’,6-diamidino-2-phenylindole (DAPI). Quantitative analysis of histological staining and fluorescence was used ImageJ software [5].

***Immunohistochemistry (IHC) and apoptosis evaluation***

Apoptosis was detected in fixed lung sections with anti-cleaved caspase-3 antibody. Primary antibody was applied over the sections and followed with biotinylated anti-rabbit IgG. Peroxidase-conjugated streptavidin with diaminobenzidine (DAB) performed as the substrate, completed the immunostaining. The number of cleaved caspase-3-positive cells was counted and normalized by alveolar perimeter.

***Flow cytometry for cell apoptosis***

The hPMVECs with different treatments were stained with Annexin-V and PI following the manufacturer’s protocol (R&D Systems, Minneapolis, MN). Apoptosis Inducer Kit TNF-α+SM-164 (Cat# C0006S, Beyotime Biotechnology) was used as a positive control. Apoptosis rate was quantified by flow cytometry (Beckman Coulter, Brea, CA, USA).

***Enzyme-linked immunosorbent assay (ELISA)***

The glycocalyx components (Heparan Sulfate, Chondroitin Sulfate, Syndecan-1, and Syndecan-4) of human and animal samples were detected using ELISA kit (all purchased from Shanghai Lanpai Bio. Tech. Co., Shanghai, China). Briefly, the serums of samples and the standard samples were added to the 96-well plates of ELISA kit in triplicate. Then 100 μl horseradish peroxidase (HRP)-conjugated antibody was added in each well, covered with an adhesive strip and incubated for 2 h at room temperature. Afterwards, the well was washed five times, substrate solution was added to each well, and incubated for 15 min at room temperature in the dark. Then, the stop solution was added to each well. Absorbance was determined at 450 nm using the ELX800 microplate reader (BioTek, Winooski, VT, USA) within 15 min.

***Western blot and*** ***Quantitative real-time*** ***polymerase chain reaction (PCR)***

Western blot analysis was performed as previously described [6]. The antibodies used in this study were summarized in **Supplementary Table 2**. Total RNA extraction and RT-qPCR procedure were performed as previously described [6]. The primers used in this study were summarized in **Supplementary Table 3.**

***Bioinformatical analysis***

The mRNA expression data in Gene Expression Omnibus (GEO) are publicly available from the GEO database (GSE37768 and GSE69818) [7]. The lung tissue samples from COPD patients, healthy smokers and nonsmokers were obtained from GEO database. COPD was defined based on t[he revised GOLD 2017 COPD categorization](https://www.sciencedirect.com/science/article/pii/S0954611117304110) [8]. FGFR1 and FGFR2 expression profile were shown in different subgroups, and adjusted p-value was calculated using a method of false discovery rate (FDR) procedure (Benjamini & Hochberg) applied by GEO2R statistical analysis.

To screen the overlapping genes relating to apoptosis in COPD, “GO_APOPTOTIC_SIGNALING_PATHWAY” including 1980 genes related to apoptosis was downloaded from GSEA (http://www.broadinstitute.org/gsea/index.jsp). Sakornsakolpat *et al.* identified 82 loci in association with either COPD or population-based measures of lung function, including 47 loci previously described and 35 new [9]. To further explore potential mechanisms in CSE-induced EC apoptosis and glycocalyx repair, we screened overlapping genes related both to apoptosis pathway and COPD. Finally, 4 genes (BTC, CITED2, IER3, THRA) were identified as overlapping co-related gene included in "35 new loci" and "apoptosis pathway", and 10 genes (TGFB2, SLC30A10, NR4A2, TWIST2, MECOM, HHIP, ITGA1, CFDP1, SOX9, RARB) in "47 loci previously described" and "apoptosis pathway".

To predict the combination site of SOX9 on the promoter region of HS6T1 (-2000 – 0 bp), we used the ConTra v3 web server to acquire the data from TRANSFAC and JASPAR database [10].

***Supplementary Figures***


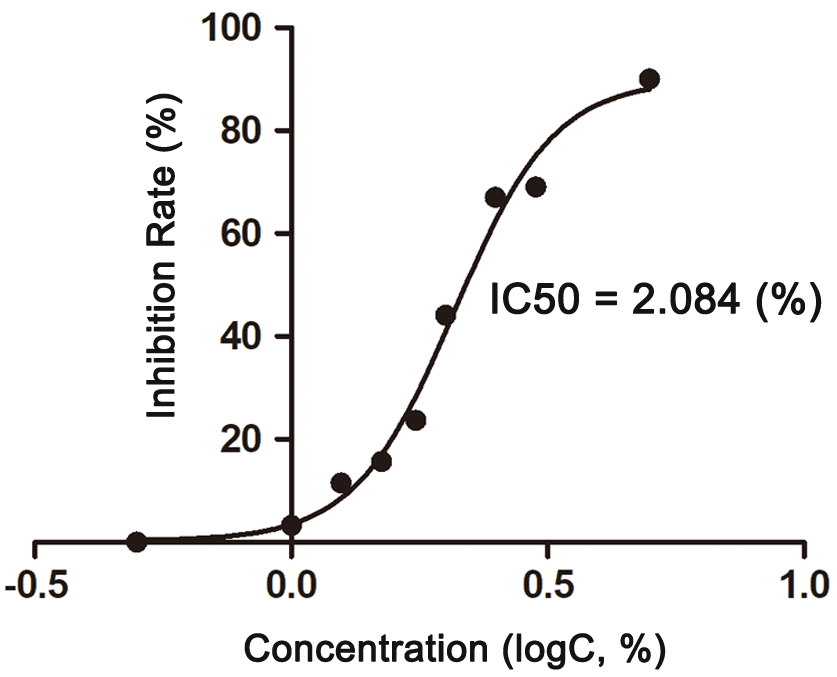


***Supplementary Figure 1. IC50 of cigarette smoke extract.*** The CCK8 assay (Dojindo Molecular Technologies, Tabaru, Japan) was used to determine cytotoxicity of CSE in hPMVECs. Cells (5000 cells/well) were seeded in a 96-well plate for each CSE concentration (0.5%-5%) and cultured for 24 h. The half maximal inhibitory concentration (IC50) of CSE to endothelial cells was determined.


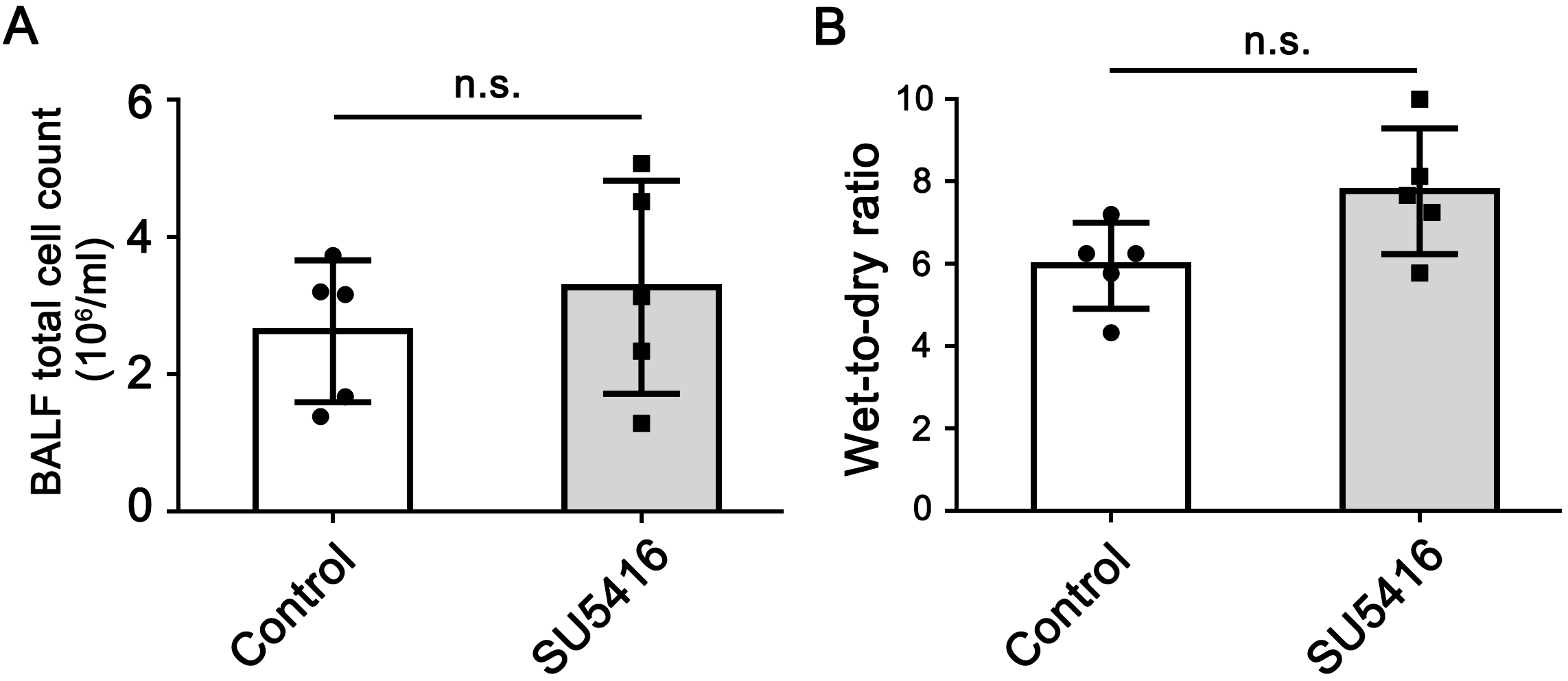


***Supplementary Figure 2. SU5416 causes a*** ***mild dysfunction in alveolar-capillary barrier and lung permeability in rats.*** SU5416-induced rat emphysema model was established, followed by bronchoalveolar lavage fluid (BALF) total cell count using a hemocytometer **(A)** and lung wet-to-dry ratio measurement **(B)**. For experiments in which lung wet/dry ratio was measured, after blood collection, the entire unravaged lung was extracted and immediately weighed (wet weight) and weighed again after being placed in a 60°C drying oven for 24 hours (dry weight) (n=5). *n.s.*, not significant.


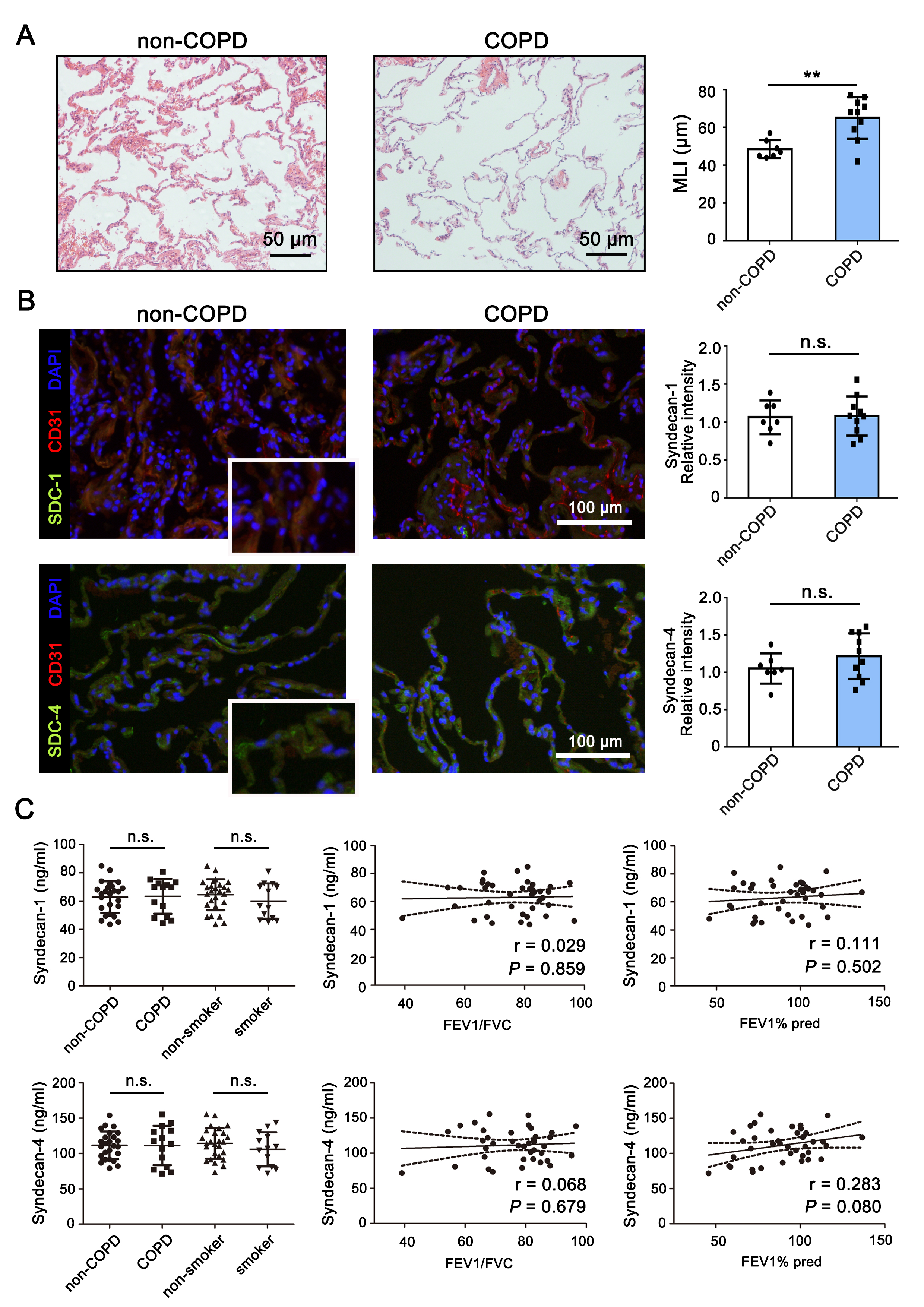


***Supplementary Figure 3.*** ***Syndecan-1 and Syndecan-4 are not impaired in COPD patients.* (A)** Representative haematoxylin and eosin (H&E) staining images of airspace. Alveolar size was measured by mean linear intercept (MLI) (n=7–10). Scale bar = 50 μm. **(B)** Immunofluorescence staining of Syndecan-1 and Syndecan-4 in non-COPD and COPD lungs. CD31 was used as the mark of endothelial cell. Scale bar = 100 μm. Quantitative analysis of fluorescence intensity for Syndecan-1 and Syndecan-4 was performed (n=7–10). **(C)** Serum Syndecan-1 and Syndecan-4 levels in subjects detected by ELISA (non-COPD n = 25, COPD n = 14, non-smoker n = 26, smoker n = 13). Correlations of circulating Syndecan-1 and Syndecan-4 levels with lung function parameters (the ratio of FEV1/FVC and FEV1% predicted) in all subjects (n = 39) were analyzed using Pearson test. *n.s.*, not significant.


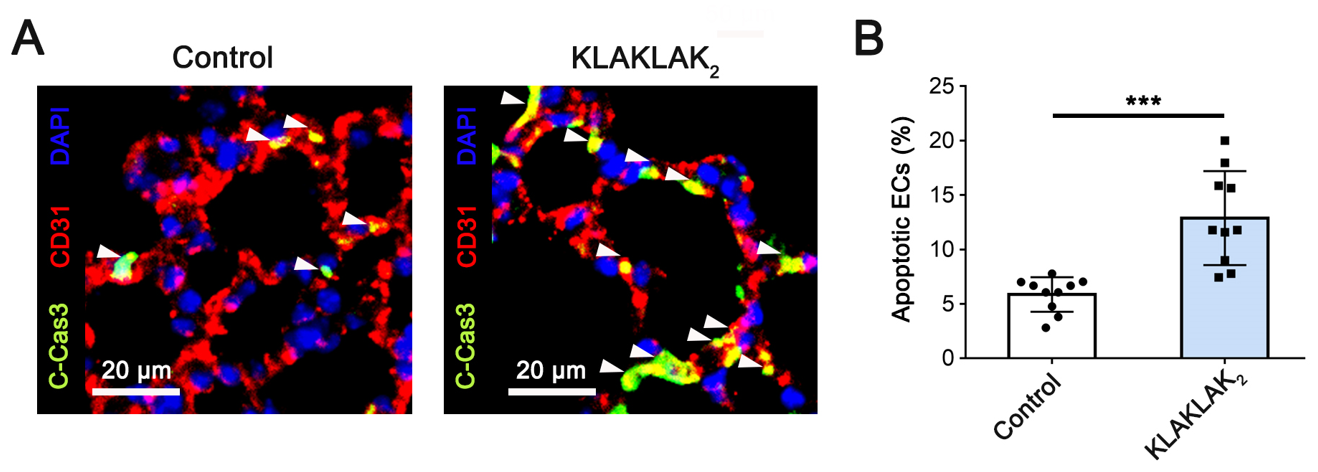


***Supplementary Figure 4. KLAKLAK2 caused endothelial apoptosis.* (A)** Representative immunofluorescence staining images of cleaved caspase-3 (white arrowheads) in mice lungs. Scale bar = 20 μm. **(B)** Quantitative analysis of percentage of apoptotic endothelial cells was performed (n=10). ****P* < 0.001.


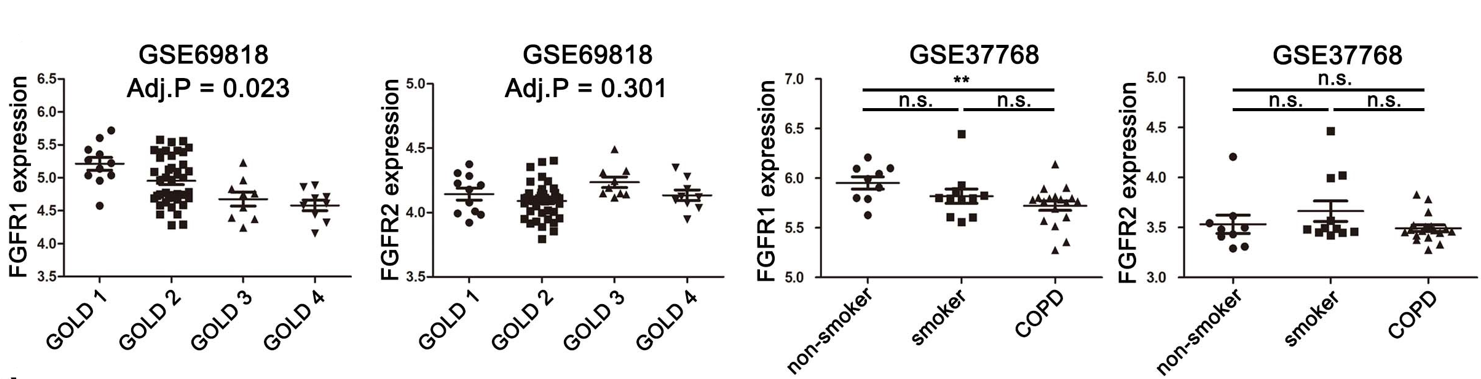


***Supplementary Figure 5. Relative FGFR1 and FGFR2 mRNA expression in COPD patients.*** The mRNA expression data in Gene Expression Omnibus (GEO) are publicly available from the GEO database (GSE37768 and GSE69818). The lung tissue samples from COPD patients, healthy smokers and nonsmokers were obtained from GEO database. FGFR1 and FGFR2 expression profile were shown in different subgroups, and adjusted p-value was calculated using a method of false discovery rate (FDR) procedure (Benjamini & Hochberg) applied by GEO2R statistical analysis.


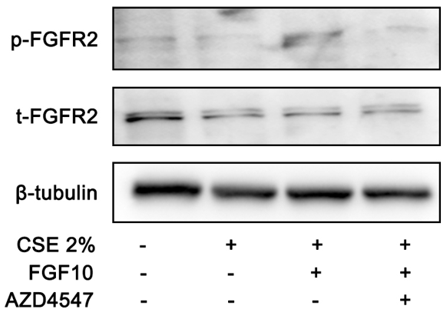


***Supplementary Figure 6.*** ***FGF10 stimulated the FGFR2 signaling in endothelial cells.*** After incubated beforehand with AZD4547 (20 nM) for 24 h, endothelial cells were then stimulated with CSE (2%) for 24 h and FGF10 (50 ng/ml) for another 15 min. DMSO was used as solvent control.


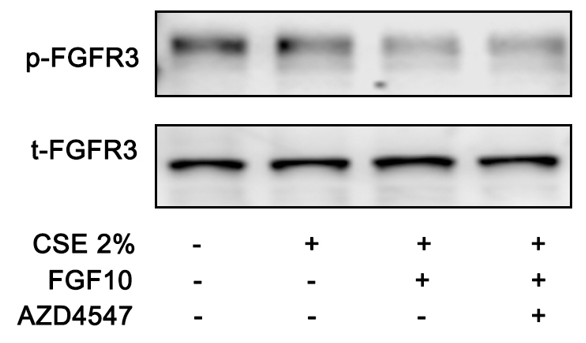


***Supplementary Figure 7. FGF10 did not stimulate the FGFR3 signaling in endothelial cells.*** After incubated beforehand with AZD4547 (20 nM) for 24 h, endothelial cells were then stimulated with CSE (2%) for 24 h and FGF10 (50 ng/ml) for another 15 min. DMSO was used as solvent control.


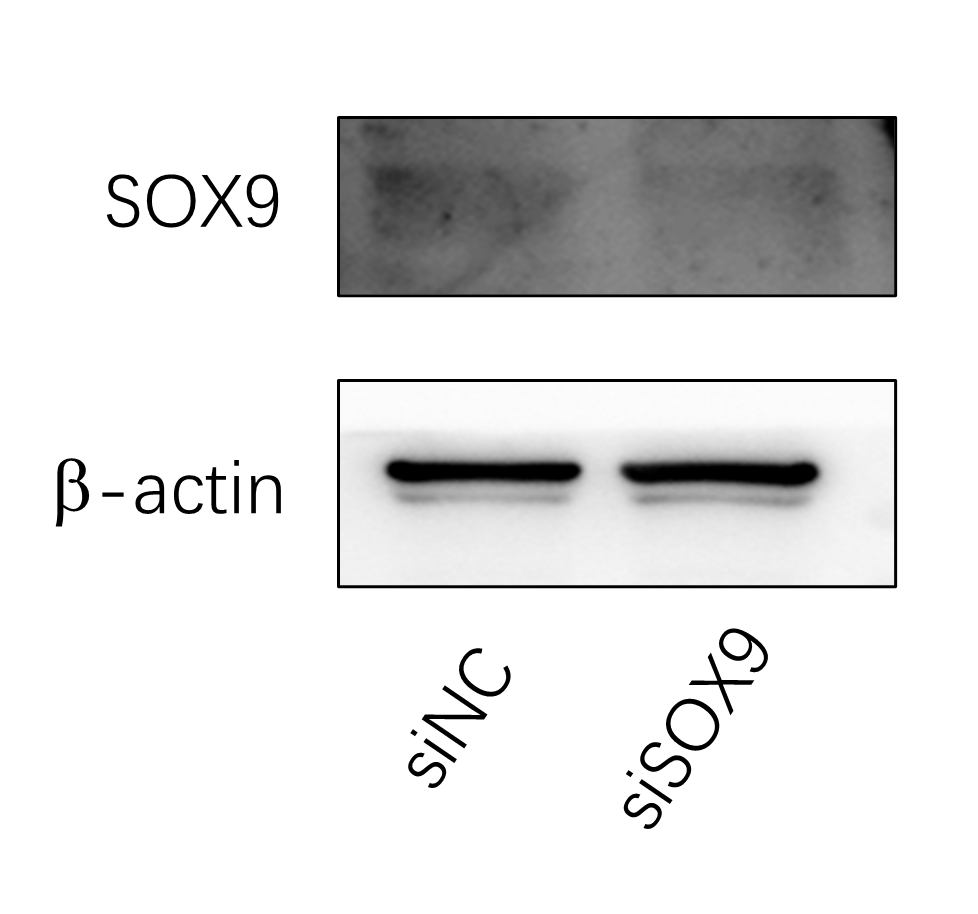


***Supplementary Figure 8.*** Endothelial cells were transfected with si-NC and si-SOX9 for 48 h and the efficacy of transfection was validated by Western blot.


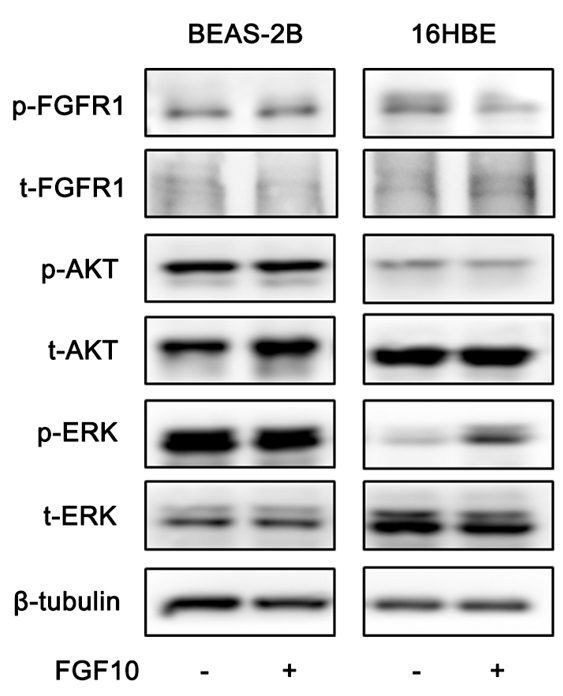


***Supplementary Figure 9. FGF10 did not stimulate the FGFR1 signaling in epithelial cells.*** BEAS-2B and 16HBE were treated with FGF10 (50 ng/ml) for 15 min. DMSO was used as solvent control.


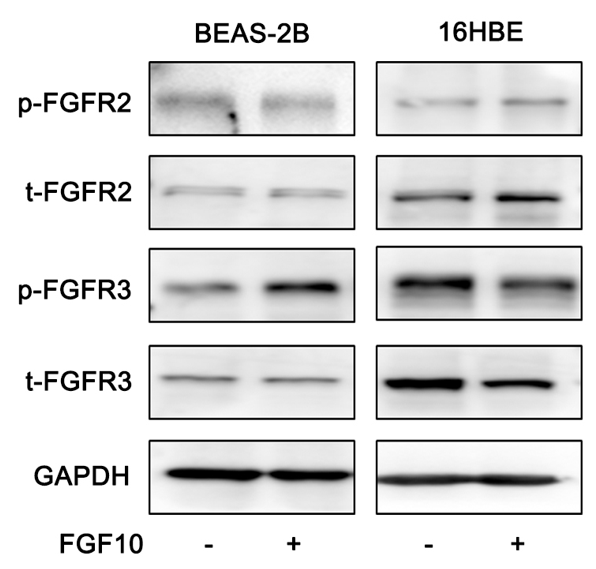


***Supplementary Figure 10. FGF10 activated the FGFR2/3 signaling in epithelial cells.*** BEAS-2B and 16HBE were treated with FGF10 (50 ng/ml) for 15 min. DMSO was used as solvent control.

***Supplementary Tables***

***Supplementary Table S1. Clinical characteristics of included subjects***

| **Variable** | **COPD (n = 14)** | **Non-COPD (n = 25)** | ***P* value** |
| --- | --- | --- | --- |
| **Age, years** | 64.79±9.893 | 60.76±11.54 | 0.2796 |
| **Sex, male/female** | 11/3 | 9/16 | <0.001 |
| **Smoking status (%)** | 7/7 | 6/19 | 0.1570 |
| **FEV1/FVC, %** | 62.73±8.29 | 83.41±5.199 | <0.001 |
| **FEV1% predicted** | 68.84±10.09 | 99.88±14.68 | <0.001 |

**NOTE:** Data are presented as mean ± SD, followed by two-sided unpaired *t*-test or *Chi*-squared test.

***Supplementary Table S2. Antibodies used in this study***

| **Antigens** | **Origin** | **Manufacturers** | **Application** |
| --- | --- | --- | --- |
| **Heparan Sulfate Proteoglycan 2 [A7L6]** | Rat | #ab2501, Abcam, Cambridge, MA, USA | 1:50-1:100 for IF |
| **Chondroitin Sulfate** **[CS-56]** | Mouse | #ab11570, Abcam, Cambridge, MA, USA | 1:50-1:100 for IF |
| **Syndecan-1** | Rabbit | #ab128936, Abcam, Cambridge, MA, USA | 2 µg/ml for IF |
| **Syndecan-4** | Rabbit | #ab24511, Abcam, Cambridge, MA, USA | 5 µg/ml for IF |
| **CD31** | Rabbit | #ab28364, Abcam, Cambridge, MA, USA | 1:50-1:100 for IF |
| **Alexa Fluor 488** | Goat Anti-rabbit | #ab150077, Abcam, Cambridge, MA, USA | 1:200 for IF |
| **Alexa Fluor 488** | Goat Anti-mouse | #ab150113, Abcam, Cambridge, MA, USA | 1:200 for IF |
| **Alexa Fluor 594** | Goat Anti-rabbit | #ab150080, Abcam, Cambridge, MA, USA | 1:200 for IF |
| **Cleaved caspase-3** | Rabbit | #ab2302, Abcam, Cambridge, MA, USA | 1:20 for IHC |
| **t****-ERK** | Rabbit | #ab17942, Abcam, Cambridge, MA, USA | 1:1000 for WB |
| **Phospho-ERK** | Rabbit | #ab201015, Abcam, Cambridge, MA, USA | 1:1000 for WB |
| **t-AKT** | Rabbit | #ab8805, Abcam, Cambridge, MA, USA | 1:1000 for WB |
| **Phospho-AKT** | Rabbit | #ab38449, Abcam, Cambridge, MA, USA | 1:1000 for WB |
| **t-FGFR1** | Rabbit | #A0082, Abclonal, Wuhan, China | 1:500 for WB |
| **Phospho-FGFR1** | Rabbit | #AP0036, Abclonal, Wuhan, China | 1:500 for WB |
| **t-FGFR2** | Rabbit | #A12436, Abclonal, Wuhan, China | 1:500 for WB |
| **Phospho-FGFR2** | Rabbit | #TA8148, Abmart, Wuhan, China | 1:500 for WB |
| **t-FGFR3** | Rabbit | #A19052, Abclonal, Wuhan, China | 1:500 for WB |
| **Phospho-FGFR3** | Rabbit | #AP1274, Abclonal, Wuhan, China | 1:500 for WB |
| **SOX9** | Rabbit | #A19710, Abclonal, Wuhan, China | 1:500 for WB |
| **Cleaved Caspase 3** | Rabbit | #ab32042, Abcam, Cambridge, MA, USA | 1:500 for IF |
| **Anti-mouse IgG HRP-linked** | Horse | #7076, Cell Signaling Technology, USA | 1:5000 for WB |
| **Anti-rabbit IgG HRP-linked** | Goat | #7074, Cell Signaling Technology, USA | 1:5000 for WB |
| **GAPDH** | Mouse | #M20006, Abmart, Wuhan, China | 1:3000 for WB |
| **β-tubulin** | Mouse | #M20005, Abmart, Wuhan, China | 1:3000 for WB |
| **β-actin** | Rabbit | #ab8227, Abcam, Cambridge, MA, USA | 1:2000 for WB |

**NOTE:** IF, immunofluorescence; IHC, immunohistochemistry. WB, western blotting.

***Supplementary Table S3. Primers for real-time qPCR and siRNA for transfection used in this study***

|  | **Forward** | **Reverse** |
| --- | --- | --- |
| **FGFR1** | GCACATCCAGTGGCTAAAGCAC | AGCACCTCCATCTCTTTGTCGG |
| **FGFR2** | GTGCCGAATGAAGAACACGACC | GGCGTGTTGTTATCCTCACCAG |
| **SOX9** | GCAGGCGGAGGCAGAGGAG | GGAGGAGGAGTGTGGCGAGTC |
| **HS6ST1** | ACTGGACCGAGCTCACCAACTG | GTCTCGTAGCAGGGTGATGTAG |
| **IL-6** | AGACAGCCACTCACCTCTTCAG | TTCTGCCAGTGCCTCTTTGCTG |
| **IL-1****β** | CCACAGACCTTCCAGGAGAATG | GTGCAGTTCAGTGATCGTACAGG |
| **TGF-β** | TACCTGAACCCGTGTTGCTCTC | GTTGCTGAGGTATCGCCAGGAA |
| **β-actin** | CACCATTGGCAATGAGCGGTTC | AGGTCTTTGCGGATGTCCACGT |
| **si-SOX9 #2**  **(siRNA)** | GCAGCGACGUCAUCUCCAATT | UUGGAGAUGACGUCGCUGCTT |

***References:***

1. Sato K, Inoue S, Igarashi A, Tokairin Y, Yamauchi K, Kimura T, Nishiwaki M, Nemoto T, Nakano H, Sato M, et al: **Effect of Iron Deficiency on a Murine Model of Smoke-induced Emphysema.** *Am J Respir Cell Mol Biol* 2020, **62:**588-597.

2. She J, Goolaerts A, Shen J, Bi J, Tong L, Gao L, Song Y, Bai C: **KGF-2 targets alveolar epithelia and capillary endothelia to reduce high altitude pulmonary oedema in rats.** *J Cell Mol Med* 2012, **16:**3074-3084.

3. Yang Y, Haeger SM, Suflita MA, Zhang F, Dailey KL, Colbert JF, Ford JA, Picon MA, Stearman RS, Lin L, et al: **Fibroblast Growth Factor Signaling Mediates Pulmonary Endothelial Glycocalyx Reconstitution.** *Am J Respir Cell Mol Biol* 2017, **56:**727-737.

4. Zhou JS, Li ZY, Xu XC, Zhao Y, Wang Y, Chen HP, Zhang M, Wu YF, Lai TW, Di CH, et al: **Cigarette smoke-initiated autoimmunity facilitates sensitisation to elastin-induced COPD-like pathologies in mice.** *Eur Respir J* 2020, **56**.

5. Jensen EC: **Quantitative analysis of histological staining and fluorescence using ImageJ.** *Anat Rec (Hoboken)* 2013, **296:**378-381.

6. Jiang T, Lin M, Zhan C, Zhao M, Yang X, Li M, Feng M: **High-pressure artificial pneumothorax promotes invasion and metastasis of oesophageal cancer cells.** *Interact Cardiovasc Thorac Surg* 2019.

7. Tojo A, Guzman NJ, Garg LC, Tisher CC, Madsen KM: **Nitric oxide inhibits bafilomycin-sensitive H(+)-ATPase activity in rat cortical collecting duct.** *Am J Physiol* 1994, **267:**F509-515.

8. Vogelmeier CF, Criner GJ, Martinez FJ, Anzueto A, Barnes PJ, Bourbeau J, Celli BR, Chen R, Decramer M, Fabbri LM, et al: **Global Strategy for the Diagnosis, Management, and Prevention of Chronic Obstructive Lung Disease 2017 Report. GOLD Executive Summary.** *Am J Respir Crit Care Med* 2017, **195:**557-582.

9. Sakornsakolpat P, Prokopenko D, Lamontagne M, Reeve NF, Guyatt AL, Jackson VE, Shrine N, Qiao D, Bartz TM, Kim DK, et al: **Genetic landscape of chronic obstructive pulmonary disease identifies heterogeneous cell-type and phenotype associations.** *Nat Genet* 2019, **51:**494-505.

10. Kreft L, Soete A, Hulpiau P, Botzki A, Saeys Y, De Bleser P: **ConTra v3: a tool to identify transcription factor binding sites across species, update 2017.** *Nucleic Acids Res* 2017, **45:**W490-W494.
